# Comparative effectiveness of psychological treatments for depressive disorders in primary care: network meta-analysis

**DOI:** 10.1186/s12875-015-0314-x

**Published:** 2015-08-19

**Authors:** Klaus Linde, Gerta Rücker, Kirsten Sigterman, Susanne Jamil, Karin Meissner, Antonius Schneider, Levente Kriston

**Affiliations:** Institute of General Practice, Technische Universität München, Orleansstr 47, D-81667 Munich, Germany; Institute for Medical Biometry and Statistics, Medical Center - University of Freiburg, Stefan-Meier-Str. 26, D-79104 Freiburg, Germany; Institute of Medical Psychology, Ludwig-Maximilians-University Munich, Goethestr 31, D-80336 Munich, Germany; Department of Medical Psychology, University Medical Center Hamburg-Eppendorf, Martinistr 52, D-20246 Hamburg, Germany

## Abstract

**Background:**

A variety of psychological interventions to treat depressive disorders have been developed and are used in primary care. In a systematic review, we compared the effectiveness of psychological treatments grouped by theoretical background, intensity of contact with the health care professional, and delivery mode for depressed patients in this setting.

**Methods:**

Randomized trials comparing a psychological treatment with usual care, placebo, another psychological treatment, pharmacotherapy, or a combination treatment in adult depressed primary care patients were identified by database searches up to December 2013. We performed both conventional pairwise meta-analysis and network meta-analysis combining direct and indirect evidence. Outcome measures were response to treatment (primary outcome), remission of symptoms, post-treatment depression scores and study discontinuation.

**Results:**

A total of 37 studies with 7,024 patients met the inclusion criteria. Among the psychological treatments investigated in at least 150 patients face-to-face cognitive behavioral therapy (CBT; OR 1.80; 95 % credible interval 1.35–2.39), face-to-face counselling and psychoeducation (1.65; 1.27–2.13), remote therapist lead CBT (1.87; 1.38–2.53), guided self-help CBT (1.68; 1.22–2.30) and no/minimal contact CBT (1.53; 1.07–2.17) were superior to usual care or placebo, but not face-to-face problem-solving therapy and face-to-face interpersonal therapy. There were no statistical differences between psychological treatments apart from face-to-face interpersonal psychotherapy being inferior to remote therapist-lead CBT (0.60; 0.37–0.95). Remote therapist-led (0.86; 0.21–3.67), guided self-help (0.93; 0.62–1.41) and no/minimal contact CBT (0.85; 0.54–1.36) had similar effects as face-to-face CBT.

**Conclusions:**

The limited available evidence precludes a sufficiently reliable assessment of the comparative effectiveness of psychological treatments in depressed primary care patients. Findings suggest that psychological interventions with a cognitive behavioral approach are promising, and primarily indirect evidence indicates that it applies also when they are delivered with a reduced number of therapist contacts or remotely.

Systematic review registration: 01KG1012 at http://www.gesundheitsforschung-bmbf.de/de/2852.php

**Electronic supplementary material:**

The online version of this article (doi:10.1186/s12875-015-0314-x) contains supplementary material, which is available to authorized users.

## Background

Psychological interventions have a central role in the treatment of depressive disorders as an alternative to or a combination with antidepressant drugs [1–4]. A variety of therapies have been developed based on cognitive-behavioural, interpersonal, psychodynamic, or humanistic approaches. A recent large network meta-analysis of 198 randomized trials in patients with depression [5] found that while the amount and the robustness of evidence varied across the single therapies the clinical effects seemed to be similar in size. Most of the trials included in this large meta-analysis were performed in specialized mental health care settings. In relation to treatment of depression in primary care two main questions arise. First, can we extrapolate the findings from trials in specialized settings to primary care? Patients with depression in primary care sometimes have less severe [6–8] and more somatic symptoms [9, 10] than patients referred to specialty mental health care. Second, the limited number and the regional distribution of qualified professionals make it difficult to provide personalized multi-session face-to-face psychological therapies on a population-wide level. Therefore, a number of interventions have been developed in which the contact time with health care professionals is reduced and/or in which the treatment is delivered by telephone, electronically, or by using printed materials. It is crucial to know how these less resource intensive methods of providing psychological treatments compare to the more intense “traditional” interventions.

We recently reported a systematic review and meta-analysis of 30 randomized trials comparing psychological treatments with usual care or placebo controls in depressed primary care patients [11]. Psychological treatments were superior to usual care. Effects of telephone- or internet-based and of reduced minimal contact cognitive behavioural approaches were broadly similar to those of personalized therapies. However, as trials had to include a usual care or placebo control group in that analysis, we excluded trials and contrasts comparing active treatments (psychological therapy, pharmacotherapy, or combination of both) with each other. Furthermore, indirect comparison of effect sizes derived from conventional meta-analyses of trials with usual care controls is methodologically problematic [12]. Network meta-analysis provides an approach to estimate effect sizes for all possible pairwise comparisons whether or not they have been compared head to head in trials making efficient use of all available evidence [13]. Thus, in the current study we utilized considerably more data than in our previous review [11] and made use of the method of network meta-analysis in order to estimate the comparative effectiveness of psychological treatments formally. By doing so, we were not only able to increase the precision of our previous estimates on comparisons with control treatments but also to provide effect size estimates for all pairwise comparisons and to test whether the evidence base is consistent (i.e., whether pieces of information from various sources such as direct and indirect comparisons agree with each other). Correspondingly, amending our previous dataset [11] with head to head comparisons, here we report a network meta-analysis of randomized trials in primary care patients with depression to compare the effectiveness of psychological treatments grouped by theoretical background, intensity of contact with the health care professional, and delivery mode.

## Methods/design

### Protocol

Details of the methods have been described in our published protocol [14] and in our conventional meta-analysis [11]. Within the overall project we also reviewed trials comparing antidepressant drugs among each other or with placebo [15].

### Literature search, study selection, assessment of risk of bias, and data extraction

We searched Medline, Embase, Cochrane Central Register of Controlled Trials (CENTRAL) and PsychINFO (main search June 2011, last update searches December 2013; see on-line supplemental material, section 1.1, for the complete Medline search strategy (Additional file 1)). We searched trial registries for unpublished and ongoing studies, and screened published systematic reviews focusing on primary care studies on depression treatments [16–19] for additional trials.

A single reviewer screened search hits and excluded clearly irrelevant records. Two reviewers independently checked all remaining records against inclusion criteria. Disagreements were resolved by discussion. We included randomized controlled trials that compared psychological or combined psychological and pharmacological interventions with one another, a pharmacological intervention, usual care or placebo in the treatment of adult primary care patients suffering from prevalent or incident unipolar depressive disorders. Psychological treatments were defined as interventions that are based on a scientific theoretical background and use psychological techniques to reduce symptoms and improve general well-being through modifying motivational, emotional, cognitive, behavioural or interpersonal processes. For inclusion, they needed to be performed either as tailored verbal communication process between a patient (or a group of patients) and a health care professional in direct or remote (e.g., per phone) contact or as a less or non-guided intervention using written information material (e.g., a book) or a computer program that the patient worked through more or less independently. Patients had to be recruited through direct referral from a primary care physician, or by systematic screening of patients in the waiting room or listed in a primary care provider’s practice. Trials had to report results on at least one of the following outcomes: response to treatment, remission of symptoms, mean score on a depression scale (post-treatment or change from baseline), or study discontinuation.

Information on patients, methods and results of all included studies were extracted by at least two independent reviewers using a pre-tested form. The Cochrane tool for assessing risk of bias was used to assess internal validity [20]. As the included studies reported results on efficacy in a very diverse and often incomplete manner, we performed an additional extraction round for extracting or imputing outcome data (see on-line supplemental material, section 1.2 for how outcomes were selected (Additional file 1)). This additional extraction was done by one experienced reviewer, while a second experienced reviewer cross-checked all extracted data and re-calculated imputations.

### Classification of treatments

We classified treatments consistently with our previous work [11] (pages 58–59): “As psychological treatments are considered complex interventions [21], grouping them can be performed along several dimensions and remains controversial [22]. Our classification system was largely pre-specified and followed published models [5], but needed some modification to account for the clinical heterogeneity in the identified primary studies. We grouped interventions according to the following dimensions: (1) theoretical background: cognitive behavioural therapy (CBT) vs. problem solving therapy (PST) vs. interpersonal therapy vs. psychodynamic therapies vs. other interventions; (2) intensity of contact with health care professional: intensively therapist-lead (with a minimum of six sessions) vs. guided self-help (with less than six sessions with the therapist) vs. no or minimal contact (with less than 90 minutes contact) interventions; and (3) face-to-face vs. remote contact interventions. Although not all dimensions of this classification system are completely independent and not all possible combinations present realistic alternatives, we considered it both comprehensive and sophisticated enough to describe reasonably differentiated treatment options that may be present in everyday care and may be relevant to health policy decision-making concerning primary care patients with depression.” Pharmacological treatments were categorized into substance classes, while usual care and placebo treatment were considered as reasonably comparable reference treatments (for example, supported by [5]).

### Outcomes

The pre-specified primary efficacy endpoint was response to treatment (an at least 50 % score reduction on a depression scale from baseline). Secondary outcomes were remission (defined as having a symptom score below a fixed threshold) and post-treatment depression scores. Study discontinuation was used as an indicator of acceptability. For participants with missing data, imputed estimates were used as reported by the trial authors. If imputed estimates were unavailable, participants with missing data were considered non-responders. In cases where responder and remission data was not reported, it was imputed from means and standard deviation using the method described by Furukawa et al. [23]. For response imputation, first the threshold of at least 50 % score reduction after completion of treatment was defined by halving the mean baseline score. Then, the proportion of participants reaching this threshold was calculated assuming a normal distribution of the data after completion of treatment. Further details, examples, and empirical justification are given by Furukawa et al. [23] and Meister et al. [24]. For our main analysis, we used data after completion of treatment.

### Statistical analyses

Due to technical reasons (some of the applied software were being developed parallel to the project), we used both Bayesian and frequentist methods. Although these two approaches have different philosophical backgrounds (for example, in the Bayesian view probability is the subjective plausibility of an event, while in the frequentist view it is the frequency of an event in multiple repeated trials), under the here applied conditions they largely agree in their findings, so that the hereby introduced methodological diversity is unlikely to be a considerable limitation [25]. For binary outcomes, we used the odds ratio as the effect measure. Conventional meta-analyses of pairwise direct comparisons within studies were performed using the inverse variance weighted random effects model option in RevMan5. For network meta-analyses of odds ratios (software WinBUGS and R interface R2WinBUGS [http://www.r-project.org/]), a Bayesian framework following the recommendations of the Decision Support Unit of the National Institute of Health and Clinical Excellence (NICE) was used to combine direct and indirect evidence [26]. Precisely, we used the code provided in Example 1(c) of [26] with a vague uniform (0.5) prior for the between-study standard deviation. For network meta-analysis of standardized mean differences (SMD), a frequentist method was applied using the R package netmeta [http://www.r-project.org/] [27, 28], using the generalized method-of-moments estimator of between-study heterogeneity given in [29]. We also calculated a generalized I^2^ statistic [30].

For a network meta-analysis to be valid, three assumptions should be met [12, 13]: 1) among trials available for head-to-head comparison study findings should be sufficiently homogeneous for each intervention group (homogeneity assumption); 2) effect estimates derived from different sources of evidence (e.g., from direct head-to-head comparisons and from indirect comparisons) should be consistent (consistency assumption); and 3) trials should be clinically sufficiently comparable (transitivity assumption). Homogeneity and consistency were investigated using a net heat plot [27]. In addition, we compared the results of the consistency model with that of an inconsistency model (the unrelated mean effects model) using the deviance information criterion (DIC) as a goodness of fit index [31]. The inconsistency model differs from the consistency model primarily in that it does not require that effect estimates for the same comparison from different evidence sources are equal (while the consistency model does). If this model fits the data considerably better than the consistency model (for example, based on the comparison of the DIC values), it can be concluded that the consistency assumption (see above) is not fulfilled. Transitivity was assessed considering clinical and methodological aspects following the recommendations by Salanti [13]. The potential impact of six pre-specified (risk of bias, recruitment method, diagnostic subtype of depression, mean age of participants, duration of treatment) and one post hoc defined covariate (sample size) was analyzed using a meta-regression model [32]. Funnel plots were produced for all direct comparisons with data from at least five trials. As there were no direct comparisons with ten or more trials, we did not perform statistical tests to distinguish chance from real asymmetry (insufficient power of such tests).

## Results

Results from 37 studies (reported in 44 publications) with 7,024 patients were included in the review (see Table 1, on-line supplemental material section 2 for references and section 3 for trial flow chart and characteristics of individual studies (Additional file 1)). 19 trials recruited patients clinically after a diagnosis and referral from the primary care physician, 16 in a screening procedure, and two used mixed approaches. 14 trials exclusively included patients meeting diagnostic criteria for major depression, and in 16 trials patients were either not formally diagnosed according to standardized schemes or patients with a variety of depressive disorders were included. Seven trials included patients with minor depression or dysthymia. The overall risk of bias was considered low in 13, unclear in 11 and high in 13 trials.

**Table 1:** Characteristics of included studies (n = 37)

Median (min., max.) publication year	2006 (1984, 2013)
Patients	
Number of patients	
- Sum	7,024
- Median (min., max.)	143 (29, 707)
Recruitment	
- Clinical	19
- Screening	16
- Mixed	2
Restricted to patients > 55 years	5
Diagnosis	
- Major depression only	14
- Depression (mixed/not exactly specified)	16
- Mild/minor/subthreshold depression and/or dysthymia	7
Overall risk of bias	
- High (high risk in one or more items)	13
- Unclear (no item high risk, < 3 low risk)	11
- Low (at least 3 low, none high risk)	13
Interventions	
Psychological interventions	
- Face-to-face cognitive behavioural psychotherapy (CBT; ≥ 6 sessions)	9
- Face-to-face other problem-solving treatment (PST; ≥ 6 sessions)	5
- Face-to-face interpersonal psychotherapy (≥ 6 sessions)	3
- Face-to-face psychodynamic therapy (≥ 6 sessions)	1
- Other face-to-face psychosocial interventions (≥ 6 sessions)	8
- Remote therapist-led CBT (≥ 6 sessions)	4
- Remote therapist-led PST (≥ 6 sessions)	2
- Guided self-help CBT (up to 4 contacts)	4
- No/minimal contact CBT (less than 30 minutes contact)	4
Pharmacotherapy	
- Selective serotonin reuptake inhibitors (SSRI)	6
- Tricyclic antidepressants	3
- Individualized antidepressant	1
Combinations psychological interventions and pharmacotherapy	
- Face-to-face CBT + SSRI	1
- Face-to-face PST + SSRI	1
- Face-to-face interpersonal therapy + SSRI	1
Contol interventions	
- Placebo	3
- Usual care	27
Median length of treatment in weeks (min., max.)	12 (6, 26)
Median number of treatment sessions (min., max.)	8 (0, 20)
Data available for meta-analysis	
Outcome response	34
Outcome remission	34
Outcome depression score data	36
Outcome total number of patients discontinuing the study	34

**Fig. 1:**
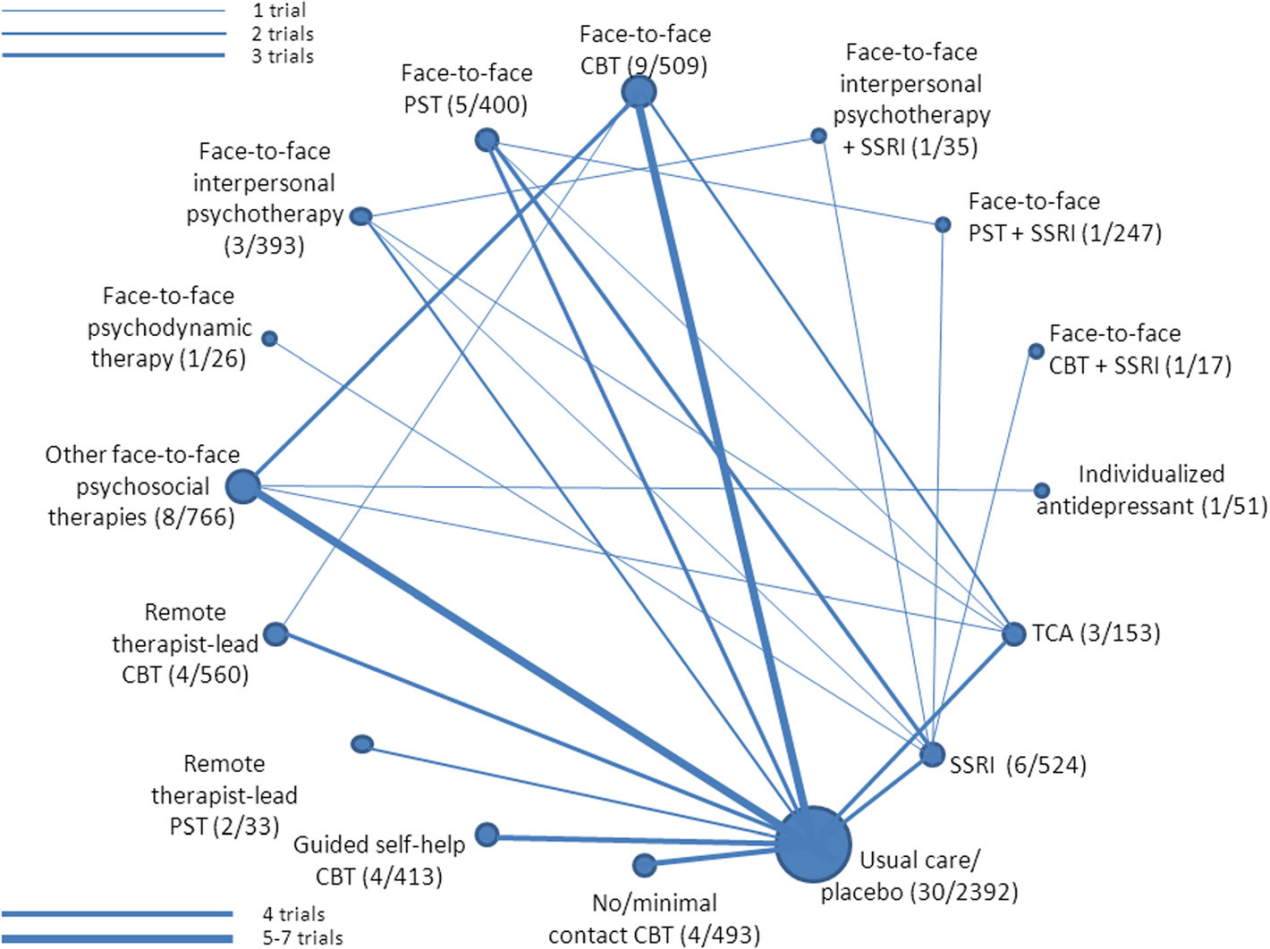
Network of available comparisons. Numbers in parentheses indicate the number of trials/number of patients in which the respective intervention was investigated. Abbreviations: CBT = cognitive behavioral therapy; PST = problem solving therapy; TCA = tricyclic and tetracyclic antidepressants; SSRI = selective serotonin reuptake inhibitors

The 37 trials had randomized patients to a total of 83 treatment arms relevant to our review (Table 1). Twenty-nine trials had two, seven trials three and one trial four relevant treatment arms. The median treatment length was 12 weeks (range 6–26 weeks). Based on our classification system interventions were allocated to 16 different classes (see Fig. 1). Forty treatment arms received a face-to-face psychological therapy with at least six treatment sessions: cognitive behavioural therapy (CBT) in nine treatment arms, problem solving therapy (PST) in five, interpersonal psychotherapy in three, psychodynamic therapy in one, and other psychological interventions in eight (seven of these used variable counselling interventions and one a psycho-educational intervention). Six treatment arms received a remote telephone or online therapist-lead intervention with at least six sessions (four trials using CBT and two using PST). In four groups, respectively, the intervention was guided self-help CBT or a no or minimal contact CBT intervention. Ten groups received antidepressants (6 selective serotonin reuptake inhibitors, SSRI, 3 tricyclic antidepressants, and 1 individualized antidepressants), 3 combinations of psychological treatments (face-to-face CBT, PST, and interpersonal psychotherapy, in one group each) and selective serotonin reuptake inhibitors (SSRI), and 30 usual care or a placebo. It should be noted that only very few patients received face-to-face psychodynamic therapy (n = 26), remote therapist-lead PST (n = 33), individualized antidepressants (n = 51), a combination of face-to-face CBT and an SSRI (n = 17), or a combination of face-to-face interpersonal psychotherapy and an SSRI (n = 35). All other interventions were tested in more than 150 patients (Fig. 1). The 16 intervention categories result in 120 possible comparisons of treatment classes, but only 25 these have actually been directly investigated in clinical trials. Among these, only the comparisons of face-to-face CBT and other face-to-face therapies with usual care or placebo were investigated in five or more trials.

**Fig. 2:**
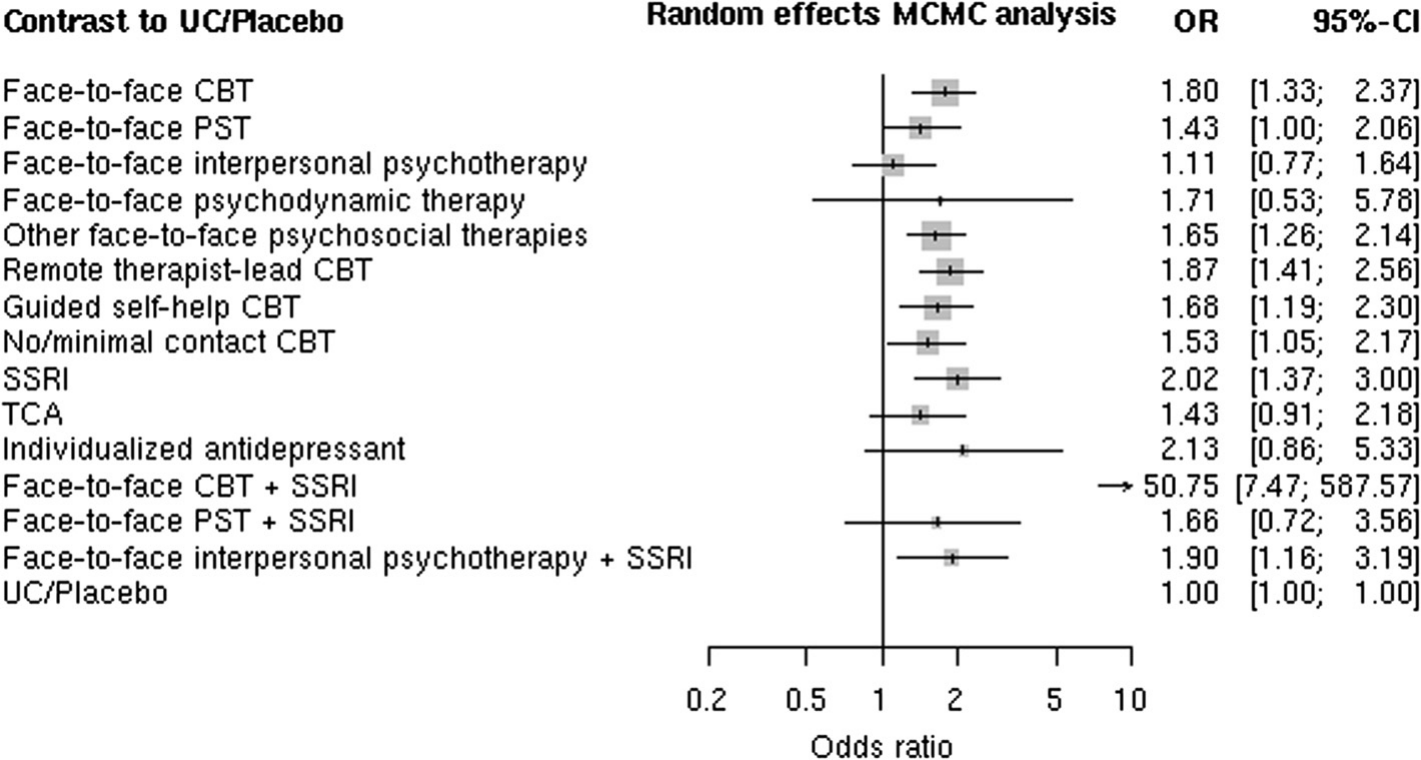
Estimates (odds ratios and 95 % credible intervals) from network meta-analysis for the primary outcome response compared to usual care/placebo control groups. MCMC = Markov chain Monte Carlo estimation [26]; OR = odds ratio. Abbreviations: CBT = cognitive behavioral therapy; PST = problem solving therapy; TCA = tricyclic and tetracyclic antidepressants; SSRI = selective serotonin reuptake inhibitors; UC = usual care

For the main outcome measure response 34 trials provided data for pairwise and network meta-analysis. These 34 trials included a total of 51 direct comparisons: 29 comparisons of a psychological treatment with usual care or placebo, 4 with another psychological treatment, 9 with drug treatment, 3 comparisons of a combination of psychological treatment and pharmacotherapy with pharmacotherapy alone, 2 of a combination treatment and a psychological treatment alone, and 4 comparisons between a drug and placebo (see Figures 4.1, 4.5, 4.9, 4.13, and 4.17 in the on-line supplementary material (Additional file 1)). Response data were not available for the two small trials investigating remote therapist-lead PST. Therefore this treatment category could not be included in this analysis. Although in conventional meta-analysis some comparisons indicated moderate statistical heterogeneity, in network meta-analysis we did not find any evidence of substantial between-trial heterogeneity, design inconsistency, or loop inconsistency (Q = 20, df = 28, p = 0.861, I^2^ = 0, DIC = 118 for consistency model, DIC = 128 for inconsistency model). The net heat plot showed only slight spots of inconsistency, mainly concerning the contrasts between face-to-face PST, TCA, and usual care (see on-line supplemental material section 7 (Additional file 1)). An inconsistency analysis, based on changes of the between-designs Q statistic following [27], located inconsistency in the comparison PST vs UC of a three-arm study [33] that showed an exaggerated effect of PST vs UC (OR 4.01; 95 % confidence interval 1.37–11.80) compared to the network estimate of 1.40 (95 % CI 0.99–1.98). Among the nine treatment categories investigated in at least 150 patients face-to-face CBT (OR 1.80; 95 % credible interval 1.35– 2.39), other face-to-face therapies (OR 1.65; 95 % CrI 1.27–2.13), remote therapist lead CBT (OR 1.87; 95 % CrI 1.38–2.53), guided self-help CBT (OR 1.68; 95 % CrI 1.22–2.30), and no/minimal contact CBT (OR 1.53; 95 % CrI 1.07–2.17) were superior to usual care or placebo, while differences were not found in the comparison of face-to-face PST and face-to-face interpersonal therapy with reference treatments (Fig. 2 and Table 2). Apart from the comparison between face-to-face interpersonal therapy and remote therapist-lead CBT (OR 0.60; 95 % CrI 0.37–0.95), there were no differences between these nine treatments. In particular, remote therapist-led (OR 0.86; 95 % CrI 0.21–0.67), guided self-help (OR 0.93; 95 % CrI 0.62–1.41) and no/minimal contact CBT (OR 0.85; 95 % CrI 0.54–1.36) had effects similar to face-to-face CBT. 95 % credible intervals of effect estimates for the five treatments tested only in single small trials were very wide. We found no differences compared to usual care or placebo for four of these (face-to-face psychodynamic therapy, individualized antidepressants, combination of face-to-face PST and SSRI and of face-to-face interpersonal psychotherapy and SSRI). The single small trial comparing the combination of face-to-face CBT and an SSRI (paroxetine or fluoxetine) with SSRI treatment alone (which was actually not taken by more than half of the patients) performed in Pakistan reported very large differences between groups. The findings of network meta-analysis based on this trial suggest that the combination of face-to-face CBT and an SSRI is much better than all other treatment categories investigated, however based on indirect evidence only and with large imprecision (credible interval). In addition, all analyses were repeated using the fixed effect model. The results were similar (not shown).

**Table 2:** Pairwise treatment comparisons from network meta-analyses for the outcomes response (main outcome measure, above the diagonal) and remission of symptoms (below the diagonal)

**Ftf CBT**	1.26	1.61	1.05	1.09	0.96	No data	1.07	1.17	**0.04**	1.08	0.94	0.89	1.26	0.84	**1.80**
	0.80–2.00	0.98–2.55	0.32–3.65	0.77–1.55	0.69–1.34		0.71–1.60	0.74–1.87	**0.00**–**0.25**	0.45–2.71	0.54–1.67	0.55–1.46	0.78–2.08	0.32–2.14	**1.33**–**2.37**
1.19	**Ftf PST**	1.28	0.84	0.87	0.76	No data	0.85	0.94	**0.03**	0.86	0.75	0.71	1.00	0.67	1.43
0.72–1.88		0.79–1.97	0.26–2.91	0.55–1.33	0.48–1.18		0.53–1.41	0.55–1.53	**0.00**–**0.19**	0.40–1.93	0.44–1.27	0.49–1.03	0.61–1.72	0.26–1.82	1.00–2.06
1.38	1.16	**Ftf IPT**	0.65	0.68	**0.60**	No data	0.66	0.73	**0.02**	0.67	**0.59**	**0.55**	0.78	0.52	1.11
0.79–2.29	0.71–1.91		0.19–2.21	0.41–1.10	**0.37**–**0.95**		0.42–1.13	0.42–1.25	**0.00**–**0.15**	0.31–1.58	**0.38**–**0.87**	**0.38**–**0.78**	0.49–1.30	0.19–1.39	0.77–1.64
0.80	0.67	0.58	**Ftf PDT**	1.04	0.91	No data	1.02	1.12	**0.03 0.00–0.31**	1.03	0.90	0.85	1.20	0.80	1.71
0.22–2.95	0.18–2.38	0.15–2.08		0.32–3.35	0.27–3.11		0.31–3.30	0.31–3.67		0.26–4.50	0.27–3.02	0.28–2.58	0.33–4.32	0.18–3.42	0.53–5.78
0.88	0.74	0.64	1.09	**Ftf Other**	0.88	No data	0.98	1.08	**0.03**	0.99	0.87	0.82	1.15	0.77	**1.65**
0.58–1.29	0.45–1.20	0.37–1.13	0.30–4.11		0.60–1.30		0.65–1.46	0.69–1.67	**0.00**–**0.22**	0.45–2.28	0.49–1.53	0.52–1.32	0.71–1.94	0.33–1.86	**1.26**–**2.14**
0.99	0.83	0.72	1.24	1.13	**Rtl CBT**	No data	1.12	1.22	**0.04**	1.13	0.98	0.93	1.31	0.88	**1.87**
0.65–1.51	0.51–1.41	0.41–1.28	0.33–4.61	0.68–1.83			0.72–1.74	0.77–2.02	**0.00**–**0.25**	0.49–2.72	0.54–1.79	0.57–1.53	0.77–2.22	0.34–2.32	**1.41**–**2.56**
1.17	0.98	0.85	1.46	1.33	1.18	**Rtl PST**	No data	No data	No data	No data	No data	No data	No data	No data	No data
0.27–4.83	0.24–4.00	0.18–3.71	0.21–9.89	0.30–5.69	0.27–5.22										
0.86	0.72	0.62	1.07	0.98	0.86	0.73	**Gsh CBT**	1.10	**0.03**	1.01	0.88	0.83	1.17	0.79	**1.68**
0.49–1.42	0.41–1.24	0.35–1.16	0.29–4.05	0.55–1.75	0.49–1.56	0.17–3.21		0.69–1.79	**0.00**–**0.22**	0.42–2.55	0.48–1.57	0.49–1.38	0.70–2.00	0.31–2.03	**1.19**–**2.30**
1.08	0.91	0.78	1.34	1.23	1.09	0.92	1.26	**Nmc CBT**	**0.03**	0.92	0.80	0.76	1.07	0.72	**1.53**
0.60–2.00	0.50–1.72	0.41–1.58	0.34–5.37	0.66–2.31	0.58–2.19	0.20–4.13	0.65–2.45		**0.00**–**0.22**	0.39–2.21	0.42–1.52	0.45–1.29	0.59–1.93	0.27–2.03	**1.05**–**2.17**
**0.08**	**0.06**	**0.05**	**0.09**	**0.09**	**0.08**	**0.06**	**0.09**	**0.03**	**Ftf CBT + SSRI**	**30.6**	**26.7**	**25.12**	**35.6**	**23.8**	**50.8**
**0.01**–**0.40**	**0.01**–**0.34**	**0.01**–**0.30**	**0.01**–**0.85**	**0.01**–**0.46**	**0.01**–**0.42**	**0.01**–**0.55**	**0.01**–**0.48**	**0.00**–**0.22**		**4.59**–**323**	**4.07**–**289**	**4.00**–**252**	**4.93**–**397**	**2.61**–**303**	**7.47**–**587**
0.99	0.83	0.72	1.23	1.13	1.00	0.85	1.15	0.92	**13.2**	**Ftf PST + SSRI**	0.87	0.82	1.16	0.78	1.66
0.35–2.27	0.35–1.91	0.26–1.75	0.27–5.57	0.41–2.67	0.36–2.33	0.17–4.06	0.44–3.02	0.39–2.21	**2.16**–**106**		0.35–2.06	0.36–1.85	0.47–3.06	0.22–2.59	0.72–3.56
0.99	0.83	0.72	1.24	1.13	1.00	0.85	1.16	0.80	**13.2**	1.00	**Ftf IPT + SSRI**	0.94	1.33	0.89	**1.90**
0.52–1.91	0.47–1.57	0.43–1.24	0.31–5.05	0.59–2.18	0.51–2.02	0.19–4.06	0.56–2.28	0.42–1.52	**2.19**–**96.9**	0.38–2.83		0.63–1.42	0.75–2.45	0.33–2.63	**1.16**–**3.19**
1.01	0.85	0.73	1.26	1.15	1.01	0.86	1.17	0.76	**13.4**	1.02	1.01	**SSRI**	1.41	0.95	**2.02**
0.61–1.64	0.57–1.23	0.48–1.12	0.37–4.43	0.70–1.91	0.60–1.74	0.20–3.72	0.68–2.03	0.45–1.29	**2.57**–**91.3**	0.47–2.43	0.57–1.69		0.84–2.50	0.37–2.54	**1.37**–**3.00**
1.03	0.86	0.75	1.28	1.17	1.04	0.88	1.20	1.07	**13.7**	1.04	1.04	1.02	**TCA**	0.67	1.43
0.59–1.75	0.50–1.42	0.45–1.25	0.35–4.75	0.66–1.97	0.59–1.83	0.21–4.35	0.66–2.26	0.59–1.93	**2.52**–**100**	0.41–2.88	0.53–1.99	0.61–1.76		0.24–1.85	0.91–2.18
0.60	0.50	0.43	0.75	0.68	0.60	0.51	0.70	0.72	**7.96**	0.61	0.60	0.59	0.58	**Individ. Antid.**	2.13
0.23–1.52	0.20–1.33	0.15–1.23	0.15–3.72	0.28–1.63	0.22–1.66	0.10–2.54	0.25–2.04	0.27–2.03	**1.18**–**74.0**	0.17–2.13	0.21–1.71	0.22–1.64	0.22–1.59		0.86–5.33
**1.53**	1.28	1.11	1.90	**1.74**	**1.54**	1.31	**1.78**	**1.53**	**20.3**	1.54	1.54	**1.51**	1.48	2.55	**UC/placebo**
**1.09**–**2.13**	0.89–1.85	0.72–1.72	0.55–6.69	**1.21**–**2.53**	**1.07**–**2.29**	0.31–5.44	**1.15**–**2.65**	**1.05**–**2.17**	**3.8**–**161**	0.69–3.96	0.86–2.76	**1.05**–**2.23**	0.92–2.39	1.00–6.67	

With regard to response, odds ratios higher than 1 favor the row-defining treatment, and odds ratios lower than 1 favor the column-defining treatment. With regard to remission, odds ratios higher than 1 favor the column-defining treatment, and odds ratios lower than 1 favor the row-defining treatment. Reciprocals should be used to obtain odds ratios in the opposite direction. Bold-faced figures indicate a difference between treatments with 95 % credible intervals of estimates excluding 1

Abbreviations: CBT = cognitive behavioral therapy; Ftf = face-to-face; Gsh = guided self-help; IPT = interpersonal therapy; Nmc = no/minimal contact; PDT = psychodynamic therapy; PST = problem solving therapy; Rtl = remote therapist-lead; SSRI = selective serotonin reuptake inhibitors; TCA = tricyclic and tetracyclic antidepressants; UC = usual care

The results are odds ratios with 95 % credible intervals when comparing the column-defined treatments with the row-defined treatments

In meta-regression analyses, recruitment method, age group, risk of bias, sample size, length of treatment in weeks, and number of sessions did not moderate treatment effects, while effects were smaller in trials in patients with minor depression and/or dysthymia (see on-line supplemental material section 6 (Additional file 1)). Findings were broadly similar when remission or post-treatment depression scores were used as outcome measures instead of response (Table 2 below and Table 3 above the diagonal; see on-line supplementary section 4 for direct comparisons (Additional file 1)). Visual inspection of funnel plots (see on-line supplemental material section 5 (Additional file 1)) suggested that smaller trials tended to report more positive findings for psychological treatments compared usual care/placebo and antidepressant drugs. However, as the number of trials per treatment category was very small funnel plots were not analysed using statistical methods.

**Table 3:** Pairwise treatment comparisons from network meta-analyses for the outcomes post-treatment depression scores (above the diagonal) and study discontinuation (below the diagonal)

**Ftf CBT**	0.18	0.21	0.00	0.02	−0.02	−0.16	−0.04	0.06	**−1.31**	−0.17	0.08	0.05	0.11	−0.02	**0.34**
	−0.09–0.44	−0.07–0.50	−0.66–0.67	−0.21–0.25	−0.25–0.20	−0.88–0.55	−0.31–0.22	−0.20–0.33	**−2.15**–**(−0.47)**	−0.69–0.35	−0.29–0.46	−0.22–0.32	−0.19–0.41	−0.56–0.53	**0.16**–**0.51**
1.47	**Ftf PST**	0.04	−0.17	−0.16	−0.20	−0.34	−0.22	−0.11	**−1.48**	−0.35	−0.09	−0.13	−0.06	−0.19	0.16
0.65–3.51		−0.23–0.31	−0.81–0.47	−0.42–0.11	−0.48–0.08	−1.06–0.38	−0.50–0.06	−0.39–0.17	**−2.31**–**(−0.66)**	−0.81–0.12	−0.44–0.25	−0.34–0.09	−0.36–0.24	−0.76–0.37	−0.04–0.36
0.98	0.67	**Ftf IPT**	−0.21	−0.19	−0.24	−0.38–1.11–	−0.26	−0.15	**−1.52**	−0.38	−0.13	−0.16	−0.10	−0.23	0.12
0.30–3.11	0.25–1.70		−0.86–0.44	−0.48–0.09	−0.54–0.06	0.35	−0.56–0.05	−0.45–0.15	**−2.36**–**(−0.69)**	−0.90–0.14	−0.44–0.17	−0.41–0.08	−0.40–0.19	−0.81–0.34	−0.11–0.35
1.72	1.17	1.75	**Ftf PDT**	0.02	−0.03	−0.17	−0.05	0.06	**−1.31**	−0.17	0.08	0.05	0.11	−0.02	0,33
0.24–15.2	0.18–9.32	0.25–14.41		−0.65–0.68	−0.70–0.64	−1.11–0.78	−0.72–0.62	−0.61–0.73	**−2.31**–**(−0.31)**	−0.95–0.60	−0.60–0.75	−0.56–0.65	−0.57–0.79	−0.85–0.81	−0,31–0,97
0.99	0.67	1.01	0.58	**Ftf Other**	−0.04	−0.19	−0.06 –0.33–	0.04	**−1.33**	−0.19	0.06	0.03	0.09	−0.04	**0.31**
0.48–2.12	0.27–1.59	0.34–3.07	0.07–4.46		−0.30–0.21	−0.90–0.53	0.20	−0.22–0.30	**−2.17**–**(−0.49)**	−0.71–0.33	−0.31–0.43	−0.24–0.30	−0.20–0.39	−0.54–0.46	**0.14**–**0.49**
1.75	1.19	1.79	1.02	1.77	**Rtl CBT**	−0.14	−0.02	0.09	**−1.29**	−0.15	0.11	0.07	0.14	0.01	**0.36**
0.67–4.46	0.36–3.51	0.50–6.34	0.09–7.89	0.59–5.14		−0.86–0.58	−0.30–0.26	−0.19–0.36	**−2.13**–**(−0.44)**	−0.68–0.38	−0.28–0.49	−0.21–0.36	−0.18–0.45	−0.55–0.57	**0.17**–**0.55**
0.74	0.51	0.76	0.43	0.75	0.42	**Rtl PST**	0.12	0.23	**−1.14**	0.00	0.25	0.22	0.28	0.15	0.50
0.17–3.23	0.11–2.22	0.15–3.32	0.04–4.66	0.18–3.21	0.08–2.17		−0.60–0.84	−0.49–0.95	**−2.22**–**(−0.07)**	−0.86–0.85	−0.52–1.02	−0.51–0.94	−0.46–1.02	−0.72–1.02	−0.19–1.19
0.62	0.42	0.63	0.36	0.63	0.35	0.83	**Gsh CBT**	0.11	**−1.26**	−0.13	0.13	0.09	0.16	0.03	**0.38**
0.24–1.46	0.16–1.00	0.20–1.88	0.04–2.81	0.25–1.56	0.11–1.08	0.19–3.80		−0.17–0.39	**−2.11**–**(−0.42)**	−0.66–0.41	−0.26–0.51	−0.20–0.38	−0.17–0.48	−0.54–0.59	**0.18**–**0.58**
0.66	0.45	0.68	0.39	0.67	0.38	0.89	1.07	**Nmc CBT**	**−1.37**	−0.23	0.02	−0.01	0.05	−0.08 –0.64–	**0.27**
0.21–2.08	0.15–1.37	0.19–2.16	0.04–3.48	0.23–1.96	0.102–1.43	0.18–4.43	0.35–3.46		**−2.22**–**(−0.53)**	−0.76–0.30	−0.37–0.40	−0.30–0.2	−0.27–0.37	0.48	**0.07**–**0.47**
No data	No data	No data	No data	No data	No data	No data	No data	No data	**Ftf CBT + SSRI**	**1.14**	**1.39**	**1.36**	**1.42**	**1.29**	**1.64**
										**0.21**–**2.07**	**0.54**–**2.24**	**0.56**–**2.16**	**0.57**–**2.27**	**0.31**–**2.27**	**0.82**–**2.47**
2.04	1.39	2.08	1.19	2.06	1.16	2.74	3.29	3.07	No data	**Ftf PST + SSRI**	0.25	0.22	0.28	0.15	**0.50**
0.40–9.99	0.34–5.61	0.40–10.65	0.12–12.2	0.40–9.90	0.20–6.44	0.31–20.2	0.65–18.1	0.56–16.7			−0.31–0.81	−0.26–0.70	−0.26–0.82	−0.57–0.88	**0.01**–**1.00**
1.54	1.05	1.58	0.90	1.56	0.88	2.07	2.49	2.32	No data	0.76	**Ftf IPT + SSRI**	−0.03	0.03	−0.10	0.25
0.38–6.64	0.29–4.04	0.50–5.10	0.09–7.19	0.39–6.64	0.18–4.16	0.33–13.7	0.55–10.5	0.48–12.5		0.13–4.68		−0.33–0.27	−0.36–0.42	−0.72–0.53	−0.08–0.58
1.26	0.86	1.29	0.73	1.27	0.72	1.69	2.03	1.90	No data	0.62	0.82	SSRI	0.06	−0.07	**0.28**
0.52–3.18	0.44–1.76	0.53–3.20	0.11–4.10	0.51–3.45	0.22–2.39	0.39–8.14	0.74–5.83	0.60–6.77		0.16–3.11	0.26–2.73		−0.24–0.37	−0.64–0.50	**0.08**–**0.49**
1.54	1.05	1.58	0.90	1.56	0.88	2.08	2.49	2.33	No data	0.76	1.00	1.23	**TCA**	−0.13	0.22
0.45–5.36	0.37–3.10	0.57–4.37	0.09–7.67	0.50–5.56	0.22–3.54	0.40–11.0	0.70–9.17	0.70–9.57		0.13–4.10	0.23–4.25	0.39–3.90		−0.71–0.45	−0.03–0.48
2.01	1.37	2.06	1.17	2.03	1.15	2.71	3.25	3.04	No data	0.99	1.31	1.60	1.30	**Individ.. Antid.**	0.35
0.40–9.46	0.25–7.50	0.32–11.74	0.08–12.9	0.46–8.52	0.18–6.73	0.35–19.8	0.57–18.5	0.47–18.4		0.10–9.23	0.17–9.78	0.26–8.45	0.21–8.41		−0.18–0.88
1.00	0.68	1.03	0.59	1.01	0.57	1.35	1.62	1.51	No data	0.49	0.65	0.80	0.65	0.50	**UC/placebo**
0.57–1.86	0.34–1.27	0.40–2.39	0.08–4.08	0.56–1.86	0.22–1.45	0.36–5.16	0.80–3.24	0.60–3.76		0.11–2.22	0.18–2.33	0.36–1.60	0.21–1.76	0.11–2.48	

With regard to post-treatment depression scores, standardized mean differences above 0 favor the row-defining treatment, and below 0 the column-defining treatment. With regard to study discontinuation, odds ratios smaller than 1 favor the column-defining treatment, and odds ratios lower than 1 favor the row-defining treatment. Reciprocals should be used to obtain odds ratios in the opposite direction. Bold-faced figures indicate a difference between treatments with 95 % credible intervals of estimates excluding 1. For abbreviations see Table 2

The results are standardized mean differences (post-treatment depression scores with 95 % confidence intervals) and odds ratios (study discontinuation with 95 % credible intervals) when comparing the column-defined treatments with the row-defined treatments

Assessing the acceptability of treatments was possible only in a very limited manner. The number of patients experiencing adverse events or discontinuing the study due to adverse events was hardly ever reported except in studies including a drug treatment arm (and in these studies partly only for the drug groups). Study discontinuation was reported in a variable manner (drop-out from the study, treatment discontinuation, missing outcome data). Network meta-analysis did not reveal any differences between treatments, but 95 % credible intervals were wide (Table 3 below the diagonal).

## Discussion

Our findings show that while a number of randomized trials have investigated psychological treatments for depression in primary care patients it is yet difficult to decide whether some treatments should be considered more effective than others. In network meta-analyses face-to-face CBT, other face-to-face therapies (mainly counselling approaches), remote therapist lead CBT, guided self-help CBT, and no/minimal contact CBT were superior to usual care or placebo. Face-to-face PST, face-to-face interpersonal psychotherapy, face-to-face psychodynamic therapy, and remote therapist-lead PST were not different from usual care or placebo, but the last two treatments have been investigated in very few patients. With one exception (remote therapist-lead CBT was superior to face-to-face interpersonal psychotherapy), there were no differences between the single treatments, but credible intervals of effect estimates were often too wide to rule out that clinically relevant differences exist. For CBT, a variety of different delivery approaches have been investigated and the findings suggest that less resource-intensive interventions have similar effects as “traditional” face-to-face CBT.

To the best of our knowledge, this is the first network meta-analysis of primary care trials comparing different psychological treatments for depression. Compared to our conventional meta-analysis of 30 trials of psychological treatment vs. usual care [11], the network meta-analysis in this article used the available data from randomized trials in a much more comprehensive manner. In does not only include seven additional trials not having a usual care control group but also comparisons with other treatment options in trials with such a control group. For example, for the outcome response, the analysis in our standard meta-analysis covered 27 comparisons with usual care, while the network meta-analysis integrated further 24 comparisons with other treatment options. In principle, this broader evidence base should allow a better assessment of the comparative effectiveness of psychological treatment for depressive disorders. We assessed carefully whether assumptions (homogeneity, consistency and transitivity – see methods section) underlying network meta-analysis were met. The homogeneity and consistency assumptions were met sufficiently in our analyses, but due to the limited number of trials for most comparisons, power of these tests was rather low and impaired sensitivity of the investigations [34]. Statistical heterogeneity and inconsistency were low with few minor exceptions. However, we fear that the transitivity assumption is not fully met. Transitivity more or less assumes that patients could be randomized to any of the treatment arms in the network. Transitivity is violated when the treatment in question differs systematically between trials, not randomly [13]. Five of the six trials in patients with minor depression and/or dysthymia investigated problem-solving therapy. Trials limited to patients with major depression more often investigated face-to-face therapies. Studies testing a certain delivery mode might be more likely to recruit participants who are motivated to use specifically this treatment mode and not another. For example, patients preferring face-to-face psychotherapy might be less likely to embark on and comply with a no/minimal contact treatment (and vice versa). Furthermore, while most trials insufficiently reported what exactly happened in usual care groups, it seems possible that there was systematic variation here, too. We still think that performing network-analysis was justifiable, but its findings have to be interpreted with caution. The doubts regarding transitivity were a major reason for us to publish our conventional meta-analysis of pairwise comparisons with usual care controls in detail first [11]. Still, the findings of both our approaches to analyse the available database are highly consistent.

Our findings are also consistent with those of the large network meta-analysis by Barth et al. [5] including mostly trials from specialized mental health care. Effect sizes over usual care were small to moderate and differences between different psychological treatments were small to absent. This suggests that findings of trials in more specialized settings do not differ fundamentally from those of primary care trials. Our findings are also consistent with two recent conventional meta-analyses of primary care trials of psychological treatments for anxiety and depression [35, 36]. An important new feature of our review is the systematic investigation of different delivery modes. According to our meta-analysis, up to now sufficient evidence is only available for CBT, suggesting that its effectiveness is only minimally impaired by reducing the number of contacts with health care professional and by using remote administration in primary care. The formal comparisons of face-to-face with remote/limited contact CBT suggesting comparable effectiveness were sufficiently precise, largely consistent, and were based on a substantial number of trials, of which several were judged to be at a low risk of bias. However, the network meta-analytical effect estimates were almost exclusively based on indirect comparisons (mainly with usual care as the common comparator), may be somewhat biased due to possible publication bias, and might not fulfil the criterion of transitivity. In summary, although existing evidence suggests equal effectiveness of different delivery modes of CBT in primary care, confidence in this finding should remain limited until confirmed in direct head-to-head trials. The network meta-analysis by Barth et al. [5] did not find a difference between individualized face-to-face and all other delivery modes. However, this finding is difficult to interpret as an unclear number of studies using face-to-face group interventions were compared with an unclear number of pooled studies on limited or no contact interventions. The results on face-to-face interpersonal psychotherapy are somewhat discouraging showing little to no benefit over usual care unless it is combined with pharmacotherapy. This finding upholds the conflicting evidence base of this treatment including both supportive [37] and sceptical [38] pieces of information. In the present study, network meta-analytical effect estimates from three large trials comparing interpersonal therapy with active medication or usual care and from indirect comparisons were acceptably precise and consistent, but two of the trials were judged to be at high risk of bias. Thus, our global confidence in the obtained findings on interpersonal therapy in primary care was moderate and calls for further research on this treatment. The reduced effectiveness of psychological interventions in patients with dysthymia/minor depression is in agreement with current evidence [38]. However, whether this finding is the result of the minor severity of the disorder, its chronicity, or a combination of both factors remains still to be clarified.

Our network also includes three combinations of psychological face-to-face treatments and drug treatments. However, each of these combinations has been tested only in a single trial, two of which were very small. Our analyses suggest a marked superiority face-to-face CBT combined with SSRI treatment over all other treatments, but this extraordinary finding from only one small trial should be interpreted with greatest caution unless confirmed by large trials. Clearly; the available evidence does not allow any firm conclusions on the value of combination treatments in depressed primary care patients. Trials testing pharmacological treatment as sole treatment were eligible to our review only if they also included a group receiving a psychological treatment. Therefore, our analyses can provide only a crude estimate of how psychological treatments compare to pharmacotherapy.

Further research should be driven by the identified evidence gaps. In our meta-analysis, the main scaffold of the evidence base is a star-shaped network of interventions with usual care/placebo as the “golden common comparator”, with which single interventions are contrasted. Comparing active treatments in such a network relies not only strongly on the questionable transitivity assumption but also precludes reliable testing of inconsistency. Instead of performing trials that compare sufficiently investigated interventions (e.g., face-to-face CBT) with an established reference (e.g., usual care), head to head randomized comparisons of clinically heterogeneous treatments would be desirable that systematically vary not only the theoretical background but also the intensity of the contact with health care professional and the delivery mode (face-to-face vs remote, including different forms of the technical realization). This would allow for comparing the wide variety of psychological interventions in primary care without confounding of possible (self-)selection processes in the treatment-seeking population. Thus, primary care investigators of psychological interventions are encouraged to overcome the “legitimation” approach of comparing specific interventions with non-specific reference treatments and embark on the comparative effectiveness research perspective looking for answers that truly inform decision-making under routine conditions [39].

## Conclusions

Based on the limited available evidence, it is difficult to assess the comparative effectiveness of psychological treatments in depressive primary care patients with high confidence. Although any conclusion can only be tentative, findings suggest that psychological interventions with a cognitive behavioral approach are promising for the treatment of primary care patients with depression. Primarily indirect evidence with moderate confidence indicates that this is also true when the cognitive behavioral therapies are delivered with a reduced number of therapist contacts or remotely. Further large trials comparing psychological treatments with each other, with pharmacotherapy, and combinations of psychological and drug treatments under conditions of routine primary care are highly desirable.

### Availability of supporting data

The raw data used in all presented network meta-analyses is available in the forest plots in section 4 of the online supplementary material.

## Additional file

Additional file 1:
**Online supplemental material.** (DOC 812 kb)

## Abbreviations

95 % CrI: 95 % credible intervals
95 % CI: 95 % confidence intervals
CBT: cognitive behavioral therapy
CENTRAL: Cochrane Central Register of Controlled Trials
DIC: deviance information criterion
Ftf: face-to-face
Gsh: guided self-help
IPT: interpersonal therapy
Nmc: no/minimal contact
PDT: psychodynamic therapy
PST: problem solving therapy
Rtl: remote therapist-lead
OR: odds ratio
SSRI: selective serotonin reuptake inhibitors
TCA: tricyclic and tetracyclic antidepressants
UC: usual care
