# Supplementary material for: Comparative effectiveness of psychological treatments for depressive disorders in primary care: network meta-analysis
Source: BMC Fam Pract. 2015 Aug 19;16:103. doi: 10.1186/s12875-015-0314-x (PMC4545315; doi:10.1186/s12875-015-0314-x)
Supplement: Additional file 1: — Online supplemental material. (DOC 812 kb) [file 12875_2015_314_MOESM1_ESM.doc]

**Contents**

1. Literature search and preference strategy for extracting and imputing outcomes 2

2. References of included primary studies 4

3. Trial flow chart and table of included studies 7

4. Forest plots for direct comparisons 11

5. Funnel plots 26

6. Findings from meta-regression analyses 30

7. Net heat plot for investigating inconsistency 31

**1. Literature search and preference strategy for extracting and imputing outcomes**

- 1. Literature search

Main search Medline (Ovid) 8.6.2011

| [**# ▲**](http://ovidsp.tx.ovid.com/sp-3.4.1b/ovidweb.cgi?&S=AGFCFPKGOJDDABKFNCCLOBMCHJKNAA00&Sort+Sets=descending) | **Searches** | **Results** |
| --- | --- | --- |
| 1 | exp Depressive Disorder/dh, dt, pc, px, su, th [Diet Therapy, Drug Therapy, Prevention & Control, Psychology, Surgery, Therapy] | 49739 |
| 2 | exp Depression/dh, dt, pc, px, su, th [Diet Therapy, Drug Therapy, Prevention & Control, Psychology, Surgery, Therapy] | 31599 |
| 3 | (depress* or antidepress*).tw. | 271636 |
| 4 | 1 or 2 or 3 | 288675 |
| 5 | exp general practitioners/ or exp physicians, family/ or exp physicians, primary care/ | 14380 |
| 6 | exp Primary Health Care/ | 63530 |
| 7 | general practice/ or family practice/ | 58042 |
| 8 | (primary adj2 care).mp. | 79930 |
| 9 | (general practitioner* or family physician*).mp. | 38994 |
| 10 | (general practice* or family practice* or family medicine).mp. | 77213 |
| 11 | (outpatient* or out-patient*).mp. | 106092 |
| 12 | 5 or 6 or 7 or 8 or 9 or 10 or 11 | 280006 |
| 13 | (randomised controlled trial or randomized controlled trial).pt. | 306533 |
| 14 | controlled clinical trial.pt. | 82376 |
| 15 | random*.ab. | 503544 |
| 16 | placebo.ab. | 124298 |
| 17 | clinical trials as topic.sh. | 154193 |
| 18 | trial.ti. | 91920 |
| 19 | 13 or 14 or 15 or 16 or 17 or 18 | 828465 |
| 20 | exp animals/ not humans.sh. | 3598584 |
| 21 | 19 not 20 | 754266 |
| 22 | 4 and 12 and 21 | 4268 |

- 1. Preference strategy for extracting and imputing outcomes

As publications used a great variety of outcomes and sometimes reported data for more than one outcome we defined preferences which data to use.

Preference (Pref.) strategy for extraction/imputation of response data

Pref. Data to use

1 HAMD (Hamilton Rating Scale for Depression) Response

2 HAMD Response or Remission

3 MADRS (Montogomery-Asberg Depression Rating Scale) Response

4 MADRS Response or Remission

5 CGI (Clinical Global Impression) at least much improved

6 other response measures based on validated scales/instruments

7 imputation based on complete score data (following preferences 1 to 4 and 6)

8 imputation based on imputation (mostly missing SD) score data (following
 preferences 1 to 4/6)

9 other response measure

Preference strategy for extraction/imputation of remission data

1 CGI-S = 1 (normal, not ill)/CIDI and other validated diagnostic instrument = no
 depression)

2 HAMD ≤ 7 (cut-offs ± 1 point accepted)

3 MADRS ≤ 10 (cut-offs ± 1 point accepted)

4 BDI (Beck Depression Inventory) ≤ 8 (cut-offs ± 1 point accepted)

5 CGI-S = 1 or 2

6 imputation based on complete score data (following preferences 2 to 4)

7 HAMD, MADRS, BDI with other cut-offs but 6 not applicable

8 other remission criteria

Preference strategy for extraction of post-treatment depression scores

1 BDI

2 other patient self-rating scales

3 HAMD

4 MADRS
5 other professional rated scales

**2. Included primary studies**

Barret 2001
Barrett JE, Williams JW, Jr., Oxman TE, Frank E, Katon W, Sullivan M, et al. Treatment of dysthymia and minor depression in primary care: a randomized trial in patients aged 18 to 59 years. Journal of Family Practice. 2001;50(5):405-12.
Barrett JE, Williams JW, Jr., Oxman TE, Katon W, Frank E, Hegel MT, et al. The treatment effectiveness project. A comparison of the effectiveness of paroxetine, problem-solving therapy, and placebo in the treatment of minor depression and dysthymia in primary care patients: background and research plan. General Hospital Psychiatry 1999;21(4):260-73.

Bedi 2000
Bedi N, Chilvers C, Churchill R, Dewey M, Duggan C, Fielding K, et al. Assessing effectiveness of treatment of depression in primary care: Partially randomised preference trial. British Journal of Psychiatry. 2000;177(OCT.):312-8.
Chilvers C, Dewey M, Fielding K, Gretton V, Miller P, Palmer B, et al. Antidepressant drugs and generic counselling for treatment of major depression in primary care: Randomised trial with patient preference arms. British Medical Journal 2001;322(7289):772-75.

Browne 2002
Browne G, Steiner M, Roberts J, Gafni A, Byrne C, Dunn E, et al. Sertraline and/or interpersonal psychotherapy for patients with dysthymic disorder in primary care: 6-month comparison with longitudinal 2-year follow-up of effectiveness and costs. Journal of Affective Disorders. 2002;68(2-3):317-30.

Casanas 2012
Casanas R, Catalan R, del Val JL, Real J, Valero S, Casas M. Effectiveness of a psycho-educational group program for major depression in primary care: a randomized controlled trial. BMC Psychiatry 2012;12:230.

Corney 1984
Corney RH**.** Psychological medicine. Psychol-Med-(London); 1984:47p.

Corney 2005
Corney R, Simpson S**.** Thirty-six month outcome data from a trial of counselling with chronically depressed patients in a general practice setting. Psychology & Psychotherapy: Theory, Research & Practice. 2005;78(Pt 1):127-38.
Simpson S, Corney R, Fitzgerald P, Beecham J. A randomised controlled trial to evaluate the effectiveness and cost-effectiveness of counselling patients with chronic depression. Health Technol Assess 2000;4(36):1-83.

de Graaf 2009
de Graaf LE, Gerhards SA, Arntz A, Riper H, Metsemakers JF, Evers SM, et al. Clinical effectiveness of online computerised cognitive-behavioural therapy without support for depression in primary care: randomised trial. Br J Psychiatry. 2009;195(1):73-80.
de Graaf LE, Gerhards SA, Evers SM, Arntz A, Riper H, Severens JL, et al. Clinical and cost-effectiveness of computerised cognitive behavioural therapy for depression in primary care: design of a randomised trial. BMC Public Health 2008;8:224.

Dwight-Johnson 2011
Dwight-Johnson M, Aisenberg E, Golinelli D, Hong S, O'Brien M, Ludman E**.** Telephone-based cognitive-behavioral therapy for Latino patients living in rural areas: a randomized pilot study. Psychiatric Services. 2011;62(8):936-42.

Joling 2011
Joling KJ, Hout HP, van't Veer-Tazelaar PJ, Horst HE, Cuijpers P, Ven PM, et al. How effective is bibliotherapy for very old adults with subthreshold depression? A randomized controlled trial. American Journal of Geriatric Psychiatry. 2011:256-65.

Kessler 2009
Kessler D, Lewis G, Kaur S, Wiles N, King M, Weich S, et al. Therapist-delivered Internet psychotherapy for depression in primary care: a randomised controlled trial. Lancet. 2009;374(9690):628-34.

King 2000
King M, Sibbald B, Ward E, Bower P, Lloyd M, Gabbay M, et al. Randomised controlled trial of non-directive counselling, cognitive-behaviour therapy and usual general practitioner care in the management of depression as well as mixed anxiety and depression in primary care. Health Technology Assessment (Winchester, England). 2000;4(19):1-83.
Ward E, King M, Lloyd M, Bower P, Sibbald B, Farrelly S, et al. Randomised controlled trial of non-directive counselling, cognitive-behaviour therapy, and usual general practitioner care for patients with depression. I: Clinical effectiveness. British Medical Journal. 2000;321(7273):1383-8.

Laidlaw 2008
Laidlaw K, Davidson K, Toner H, Jackson G, Clark S, Law J, et al. A randomised controlled trial of cognitive behaviour therapy vs treatment as usual in the treatment of mild to moderate late life depression. International Journal of Geriatric Psychiatry. 2008;23(8):843-50.

Levesque 2011
Levesque DA, Van Marter DF, Schneider RJ, Bauer MR, Goldberg DN, Prochaska JO, et al. Randomized trial of a computer-tailored intervention for patients with depression. American Journal of Health Promotion 2011;26(2):77-89.

Levin 2011
Levin W, Campbell DR, McGovern KB, Gau JM, Kosty DB, Seeley JR, et al. A computer-assisted depression intervention in primary care. Psychological Medicine, 2011:1373-83.

Lynch 1997
Lynch DJ, Tamburrino MB, Nagel R**.** Telephone counseling for patients with minor depression: preliminary findings in a family practice setting. Journal of Family Practice. 1997;44(3):293-8.

Lynch 2004
Lynch D, Tamburrino M, Nagel R, Smith MK**.** Telephone-based treatment for family practice patients with mild depression. Psychological Reports. 2004;94(3 Pt 1):785-92.

MacPherson 2013
MacPherson H, Richmond S, Bland M, Brealey S, Gabe R, Hopton A, et al. Acupuncture and Counselling for Depression in Primary Care: A Randomised Controlled Trial. PLoS Medicine 2013;10(9).
MacPherson H, Richmond S, Bland JM, Lansdown H, Hopton A, Kang'ombe A, et al. Acupuncture, Counseling, and Usual care for Depression (ACUDep): study protocol for a randomized controlled trial. Trials 2012;13:209.*

Mohr 2012
Mohr DC, Ho J, Duffecy J, Reifler D, Sokol L, Burns MN, et al. Effect of telephone-administered vs face-to-face cognitive behavioral therapy on adherence to therapy and depression outcomes among primary care patients: a randomized trial. JAMA 2012;307(21):2278-85.

Mynors-Wallis 1995
Mynors-Wallis LM, Gath DH, Lloyd-Thomas AR, Tomlinson D**.** Randomised controlled trial comparing problem solving treatment with amitriptyline and placebo for major depression in primary care. BMJ. 1995;310(6977):441-5.

Mynor-Wallis 2000
Mynors-Wallis LM, Gath DH, Day A, Baker F**.** Randomised controlled trial of problem solving treatment, antidepressant medication, and combined treatment for major depression in primary care. British Medical Journal. 2000;320(7226):26-30.

Naeem 2011
Naeem F, Waheed W, Gobbi M, Ayub M, Kingdon D**.** Preliminary evaluation of culturally sensitive CBT for depression in Pakistan: findings from Developing Culturally-sensitive CBT Project (DCCP). Behavioural & Cognitive Psychotherapy. 2011;39(2):165-73.

Naylor 2010
Naylor EV, Antonuccio DO, Litt M, Johnson GE, Spogen DR, Williams R, et al. Bibliotherapy as a treatment for depression in primary care. J Clin Psychol Med Settings 2010;17(3):258-71.
Naylor EV**.** A five-minute bibliotherapy prescription as a physician-delivered treatment for depression. ProQuest Information & Learning; 2008.

Oxman 2008
Oxman TE, Hegel MT, Hull JG, Dietrich AJ**.** Problem-solving treatment and coping styles in primary care for minor depression. Journal of Consulting & Clinical Psychology. 2008;76(6):933-43.

Proudfoot 2004
Proudfoot J, Ryden C, Everitt B, Shapiro DA, Goldberg D, Mann A, et al. Clinical efficacy of computerised cognitive-behavioural therapy for anxiety and depression in primary care: randomised controlled trial. British Journal of Psychiatry. 2004;185:46-54.

Salminen 2008
Salminen JK, Karlsson H, Hietala J, Kajander J, Aalto S, Markkula J, et al. Short-term psychodynamic psychotherapy and fluoxetine in major depressive disorder: a randomized comparative study. Psychotherapy & Psychosomatics. 2008;77(6):351-7.

Schulberg 1996
Schulberg HC, Block MR, Madonia MJ, Scott CP, Rodriguez E, Imber SD, et al. Treating major depression in primary care practice. Eight-month clinical outcomes. Archives of General Psychiatry. 1996;53(10):913-9.

Scott 1992
Scott AI, Freeman CP**.** Edinburgh primary care depression study: treatment outcome, patient satisfaction, and cost after 16 weeks. BMJ. 1992;304(6831):883-7.

Scott 1997
Scott C, Tacchi MJ, Jones R, Scott J**.** Acute and one-year outcome of a randomised controlled trial of brief cognitive therapy for major depressive disorder in primary care. British Journal of Psychiatry. 1997;171:131-4.

Serfaty 2009
Serfaty MA, Haworth D, Blanchard M, Buszewicz M, Murad S, King M**.** Clinical effectiveness of individual cognitive behavioral therapy for depressed older people in primary care: a randomized controlled trial. Archives of General Psychiatry. 2009;66(12):1332-40.

Simon 2004
Simon GE, Ludman EJ, Tutty S, Operskalski B, Von Korff M**.** Telephone psychotherapy and telephone care management for primary care patients starting antidepressant treatment: a randomized controlled trial. JAMA. 2004;292(8):935-42.

Smit 2006
Smit A, Kluiter H, Conradi HJ, van der Meer K, Tiemens BG, Jenner JA, et al. Short-term effects of enhanced treatment for depression in primary care: results from a randomized controlled trial. Psychological Medicine. 2006;36(1):15-26.
Conradi HJ, de Jonge P, Kluiter H, Smit A, van der Meer K, Jenner JA, et al. Enhanced treatment for depression in primary care: long-term outcomes of a psycho-educational prevention program alone and enriched with psychiatric consultation or cognitive behavioral therapy. Psychological Medicine 2007;37(6):849-62.

Teasdale 1984
Teasdale JD, Fennell MJ, Hibbert GA, Amies PL**.** Cognitive therapy for major depressive disorder in primary care. British Journal of Psychiatry. 1984;144:400-6.

Van Schaik 2006
van Schaik A, van Marwijk H, Ader H, van Dyck R, de Haan M, Penninx B, et al. Interpersonal psychotherapy for elderly patients in primary care. American Journal of Geriatric Psychiatry. 2006;14(9):777-86.

Ward 2000
Ward E, King M, Lloyd M, Bower P, Sibbald B, Farrelly S, et al. Randomised controlled trial of non-directive counselling, cognitive-behaviour therapy, and usual general practitioner care for patients with depression. I: Clinical effectiveness. British Medical Journal. 2000;321(7273):1383-8.
King M, Sibbald B, Ward E, Bower P, Lloyd M, Gabbay M, et al. Randomised controlled trial of non-directive counselling, cognitive-behaviour therapy and usual general practitioner care in the management of depression as well as mixed anxiety and depression in primary care. Health Technology Assessment (Winchester, England) 2000;4(19):1-83.

Watkins 2012
Watkins ER, Taylor RS, Byng R, Baeyens C, Read R, Pearson K, et al. Guided self-help concreteness training as an intervention for major depression in primary care: a Phase II randomized controlled trial. Psychological Medicine 2012;42(7):1359-71.

Williams 2000
Williams JW, Jr., Barrett J, Oxman T, Frank E, Katon W, Sullivan M, et al. Treatment of dysthymia and minor depression in primary care: A randomized controlled trial in older adults. JAMA. 2000;284(12):1519-26.
Barrett JE, Williams JW, Jr., Oxman TE, Katon W, Frank E, Hegel MT, et al. The treatment effectiveness project. A comparison of the effectiveness of paroxetine, problem-solving therapy, and placebo in the treatment of minor depression and dysthymia in primary care patients: background and research plan. General Hospital Psychiatry 1999;21(4):260-73.

Williams 2013
Williams C, Wilson P, Morrison J, McMahon A, Andrew W, Allan L, et al. Guided self-help cognitive behavioural therapy for depression in primary care: a randomised controlled trial. PloS One, 2013:e52735.

**3. Trial flow chart and table of included studies**

3.1. Trial flow chart

**Screening**

**Included**

**Eligibility**

**Identification**

10,275 records after duplicates removed

10,275 records screened by a single reviewer

9,401 clearly irrelevant records excluded

100 full-text reports assessed for eligibility

874 records screened by 2 reviewers

774 further irrelevant records excluded

18,967 records identified through electronic database searching and other sources

56 full text reports excluded due to

- inadequate design (7)

- patients/setting (16)

- no relevant outcomes (3)

- inadequate intervention (2)

- irrelevant additional publication
 (25)

- protocol only (2)

- attrition > 50% (1)

37 studies (in 44 articles) included in systematic review

3.2. Table of included studies

| First author  Risk of Bias | n* | Recruit-ment | Depression diagnosis | % female  Mean age | Group 1  (sessions/provider) | Group 2 | Further groups | Week post-treatment measurement  Score | Response  Remission |
| --- | --- | --- | --- | --- | --- | --- | --- | --- | --- |
| **Face-to-face cognitive behavioural therapy (CBT)** | | | |  |  |  |  |  |  |
| Laidlaw 2008  u(luuul) | 44 | Referral | Major depression | 72  74 | CBT  (8/psychologist) | Usual care |  | 18  BDI | HRSD (i)  HRSD (i) |
| Naeem 2010  l(luull) | 34 | Referral | Depression | 74  33 | CBT+SSRI (9/psychologist) | SSRI |  | 6  HAD-D | HAD-D (i)  HAD-D (i) |
| Scott 1997  h(uuuhh) | 48 | Referral | Major depression | 67  41 | Brief CT  (6/therapist) | Usual care |  | 7  BDI | HRSD (i)  HRSD (i) |
| Serfaty 2009  l(llhul) | 137 | Mainly screening | Depression | 79  74 | CBT (≤12/therapist) | Usual care | Talking control** | 16  BDI | BDI (i)  BDI (i) |
| Smit 2006  l(llhll) | 116 | Referral | Major depression | 64  43 | CBT (10-12/therapist) | Usual care | Recurrence prevention** | 12  BDI | BDI (i)  BDI (i) |
| Teasdale 1984  h(uuhhl) | 44 | Screening | Major depression | 94  38 | Cognitive therapy (≤20/psychologist) | Usual care |  | Ca. 16  BDI (i) | HRSD  BDI |
| **Face-to-face problem solving therapy (PST)** | | | |  |  |  |  |  |  |
| Barrett 2001  u(luuuu) | 241 | Referral | Dysthymia, minor depr. | 64  44 | PST  (6/psychologist) | Placebo | Paroxetine | 11  HSCL-D (ci) | -  HDRS≤6 |
| Mynor-Wallis 1995  h(uuuhl) | 91 | Referral | Major depression | 77  37 | PST (6/trained physician) | Placebo | Amitriptyline | 12  BDI | HRSD (i)  HRSD (i) |
| Mynor-Wallis 2000  u(uuull) | 151 | Referral | Major depression | 77  35 | PST$ (6/ physicians or nurse) | Fluvoxamine/ Paroxetine | Both | 12  BDI | HRSD (i)  HRSD |
| Oxman 2008  u(llhuu) | 141 | Screening | Minor depression | 58  55 | PST  (6/counsellor) | Usual care |  | 9  MADRS | MADRS (i)  MADRS (i) |
| Williams 2000  h(llhlh) | 415 | Referral or screening | Dysthymia or minor depr. | 41  71 | PST (6/ psychologist/ counsellor) | Placebo | Paroxetine | 11  HSCL-20 (c) | HSCL-D (i)  HAMD |
| **Face-to-face interpersonal psychotherapy (IPT)** | | | |  |  |  |  |  |  |
| Browne 2002  h(luuhl) | 707 | Screening | Dysthymia | 68  42 | IPT  (≤12/psychotherapist) | Sertraline | Both | 26  MADRS | MADRS (40%)  MADRS (i) |
| Schulberg 1996  h(uuuhh) | 276 | Screening | Major depression | 83  38 | IPT(16/psychologist/ psychiatrist) | Usual care | Nortriptyline | 26  HRSD (i) | HRSD (i)  HRSD (i) |
| Van Schaik 2006  u(ulhul) | 143 | Screening | Major depression | 69  68 | I PT (10/psychologist/nurse) | Usual care |  | 26  MADRS | MADRS  MADRS |
| **Face-to-face psychodynamic therapy (PDT)** | | | |  |  |  |  |  |  |
| Salminen 2008  u(uuhll) | 51 | Referral | Major depression | 69  42 | PDT (16/psychologist/ psychiatrist | Fluoxetine |  | 16  BDI | HRSD  DSM-IV |
| **Other face-to-face psychosocial therapies** | | | |  |  |  |  |  |  |
| Bedi 2000  h(llhuh) | 103 | Referral | Major depression | 77  38 | Counselling  (6/counsellor) | Antidepressant |  | 8  BDI | BDI (i)  RDC<4 |
| Casanas 2012  l(llhul) | 231 | Referral | Major depression | 89  53 | Psychoeducation (12/nurse) | Usual care |  | 12  BDI | BDI (i)  BDI (i) |
| Corney 1984  h(huhuh) | 87 | Referral | Depression | 100  30 | Social work (unclear/ social worker) | Usual care |  | unclear  - | Improvement  - |
| Corney 2005  l(luhll) | 181 | Screening | Depression  (≥ 6 months) | 80  43 | Counselling  (6-12/counsellor) | Usual care |  | 26  BDI | BDI (i)  BDI (i) |
| MacPherson 2013  l(llhul) | 453 | Referral | Depression | 73  44 | Counselling (12/counsellors) | Usual care | Acupuncture** | 12  PHQ-9 | PHQ-9 (i)  PHQ-9 (i) |
| **Trials with both face-to-face CBT and other face-to-face psychosocial therapies** | | | | | | | | | |
| Scott 1992  u(uuuul) | 121 | Referral | Major depression | 76  32 | CBT  (10/psychologist) | Counselling  (13/social worker) | Usual care  Amitriptyline | 16  HRSD | HRSD (i)  HRSD |
| Ward 2000  l(ulhll) | 197 | Referral | Depression | 77  37 | CBT (6-12/psychologist) | Counselling  (6-12/counsellor) | Usual care | 16 | BDI (i)  BDI (i) |
| King 2000§  l(ulhll) | 130 | Referral | Depression | 71  33 | CBT (6-12/psychologist) | Counselling  (6-12/counsellor) |  | 16  BDI | BDI (i)  BDI (i) |
| **Trials with both face-to-face CBT and remote therapist-lead CBT** | | | | | | | | | |
| Mohr 2012  l(llhul) | 325 | Referral | Major depression | 78  48 | CBT  (18/psychologist) | Telephone CBT  (18/psychologist) |  | 18  HAMD | HRSD  HRSD |
| **Remote therapist-lead CBT** | | | |  |  |  |  |  |  |
| Dwight-Johnson 2011  l(llhul) | 101 | Screening | Depression | 78  40 | Telephone CBT (8/trained therapist) | Usual care |  | 12  SCL | SCL  - |
| Kessler 2009  h(llhhl) | 297 | Referral | Major depression | 68  35 | Online CBT (≤10/psychologist) | Usual care |  | 16  BDI | BDI (i)  BDI (i) |
| Simon 2004  u(llhuu) | 393 | Referral | Depression | 76  44 | Telephone CBT (8/psychotherapist) | Usual care (PCP) |  | 26  SCL-D | HSCL-D  SCL-D |
| **Remote therapist-lead problem solving therapy** | | | |  |  |  |  |  |  |
| Lynch 1997  h(uuhhu) | 29 | Screening | Minor depression | 86  48 | Telephone PST (6/trained student) | Usual care (no treatment) |  | 6  BDI (i) | -  BDI (i) |
| Lynch 2004  h(uuhhh) | 36 | Screening | Mild depression | 83  38 | Telephone PST  (6/nurse) | Usual care | Stress management** | 6  BDI | -  BDI (i) |
| **Guided self-help CBT** | | | |  |  |  |  |  |  |
| Joling 2011  u(luhul) | 170 | Screening | Sub-threshold depression | 54  81 | Bibliotherapy (3/nurse) | Usual care |  | 12  CES-D | CES-D reduction ≥ 5  CES-D≤16 |
| Proudfoot 2004  u(ulhuu) | 274 | Referral or screening | Depression | 74  44 | computerized CBT (≤80min./nurse) | Usual care |  | 9  BDI | BDI (i)  BDI (i) |
| Watkins 2012  l(llull) | 82 | Screening | Depression | 55  46 | Guided self-help concreteness training (≤4/psychologist) | Usual care | Relaxation control** | 8  BDI | HRSD (i)  HRSD (i) |
| Williams 2013  u(ulhul) | 281 | Referral | Depression | 68  42 | Guided self-help CBT (3/psychologist) | Usual care |  | 16  BDI | BDI  BDI (i) |
| **No/minimal contact CBT** | | | |  |  |  |  |  |  |
| de Graaf 2009  l(llhll) | 303 | Screening | Depression | 57  45 | Computerized CBT# | Usual care |  | 8  BDI | BDI reliable change  BDI (i) |
| Levesque 2011  h(luhhl) | 350 | Screening | Depression | 67  47 | Computer behavioural intervention acc. TTM | Usual care (no intervention) |  | 39  BDI | BDI ≤9+signif. change  BDI (i) |
| Levin 2011  l(luhll) | 191 | Referral | Depression | 77  44 | Computer CBT | Usual care |  | 6  CES-D | SCID symptoms (i)  - |
| Naylor 2010  h(luhhl) | 38 | Screening | Depression | 84  51 | Bibliotherapy | Usual care |  | 6  BDI-FS | BDI-FS (i)  BDI-FS (i) |

n* = number of patients randomized (only in groups included in analyses); ** = comparator or control group not included in analyses

Risk of bias: l = low, u = unclear, h = high; the first letter indicates the overall risk of blinding not taking blinding into account; letters in parentheses sequentially indicate the risk of bias for the items sequence generation, concealment, blinding, attrition and selective reporting

TTM = transtheoretical model; CES-D = Center for Epidemiologic Studies Depression Scale, BDI = Beck Depression Inventory, HAD-D = Hospital Anxiety and Depression Scale subscale depression, HRSD = Hamilton Rating Scale for Depression, MADRS = Montgomery Asberg Depression Rating Scale, PHQ-9 = Patient Health Questionnaire for Depression; RDC = Research Diagnostic Criteria, HSCL-D = Hopkins Symptom Checklist Depression Scale, (i) = imputed data, (c) = only change from baseline data available

§The studies designated by us King 2000 and Ward 2000 are different parts of the same study: King 2000 refers to the phase of the trial in which patients were randomized to two arms, Ward to the phase in which patients were randomized to three arms

#trial included two separate groups computerized CBT alone and in combination with UC which were pooled

&Patients in this group received also the psycho-educational depression recurrence intervention from group 3 after completing CBT, but this was after the outcome measurement used in meta-analysis

**4. Forest plots for direct comparisons**

4.1. Outcome: response – Comparison: psychosocial therapies (PST) vs. usual care/placebo (UC)


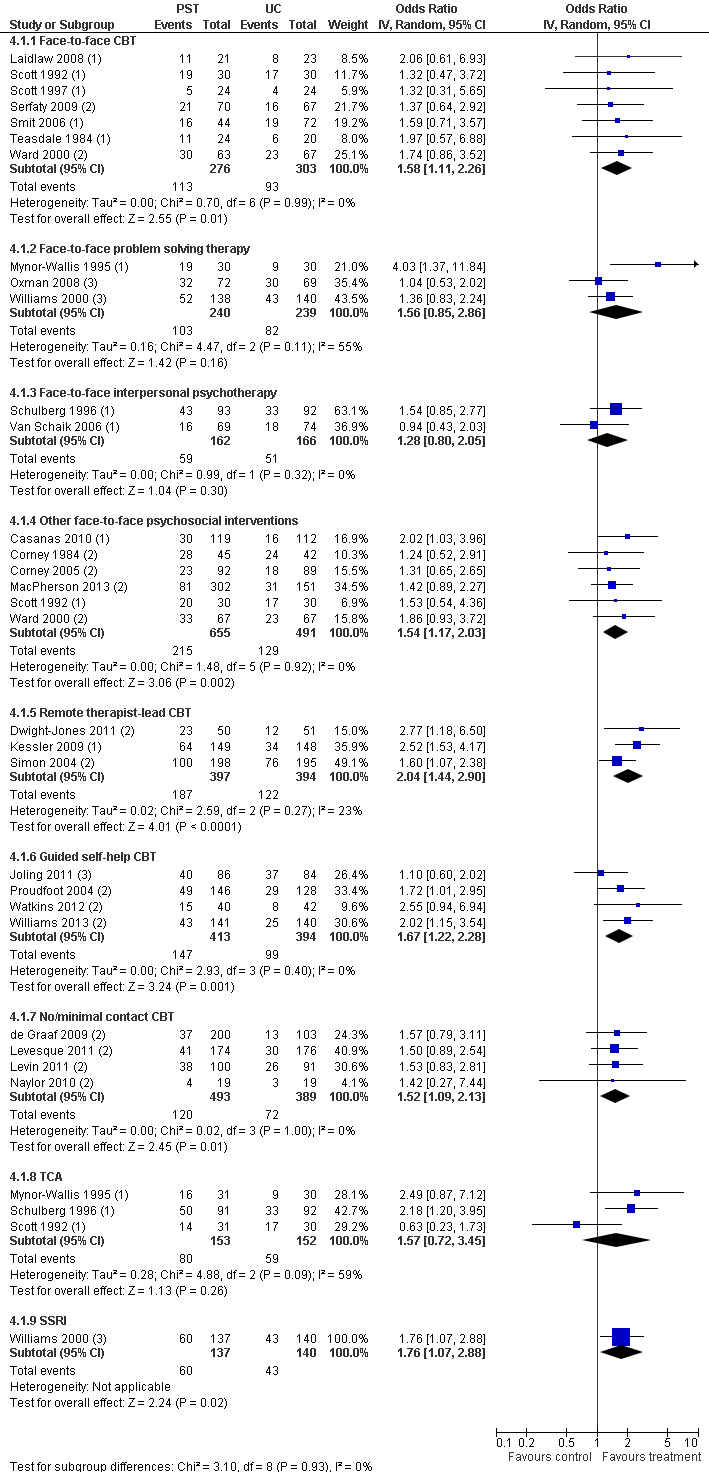


(1) = limited to patients with major depression; (2) mixed/unclear depression diagnosis; (3) minor depression/dysthymia

4.2. Outcome: remission – Comparison: psychosocial therapies (PST) vs. usual care (UC)


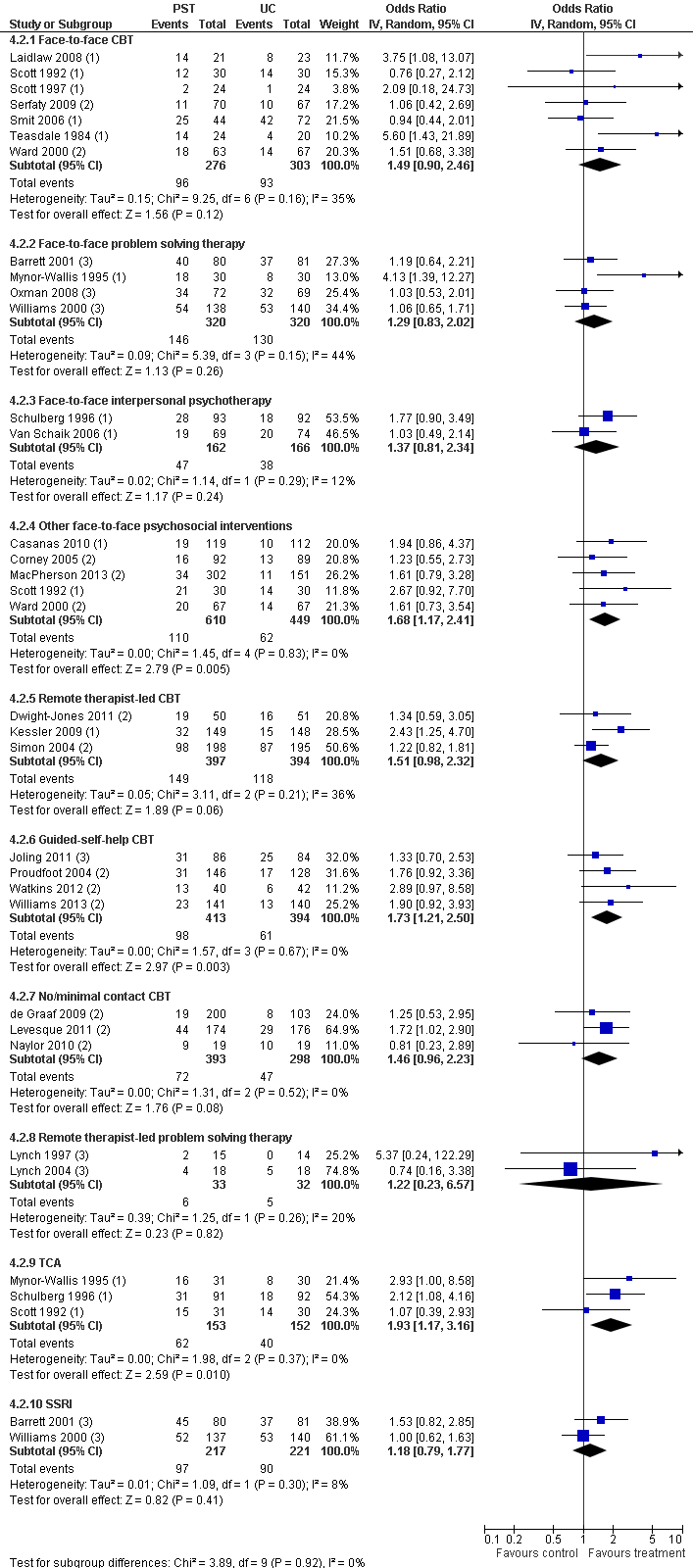


4.3. Outcome: post-treatment depression scores – Comparison: psychosocial therapies (PST) vs. usual care (UC)


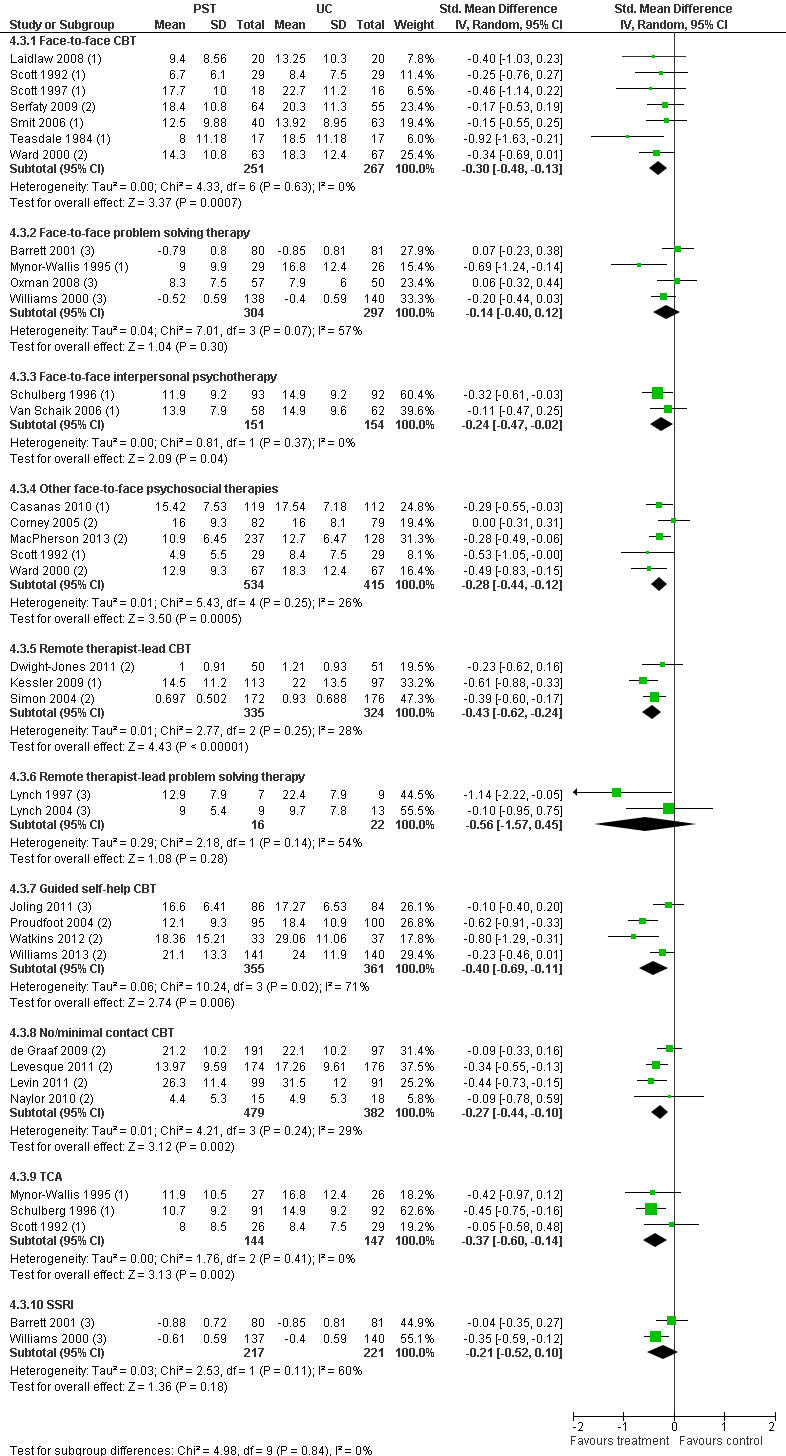


4.4. Outcome: drop-out – Comparison: psychosocial therapies (PST) vs. usual care (UC)


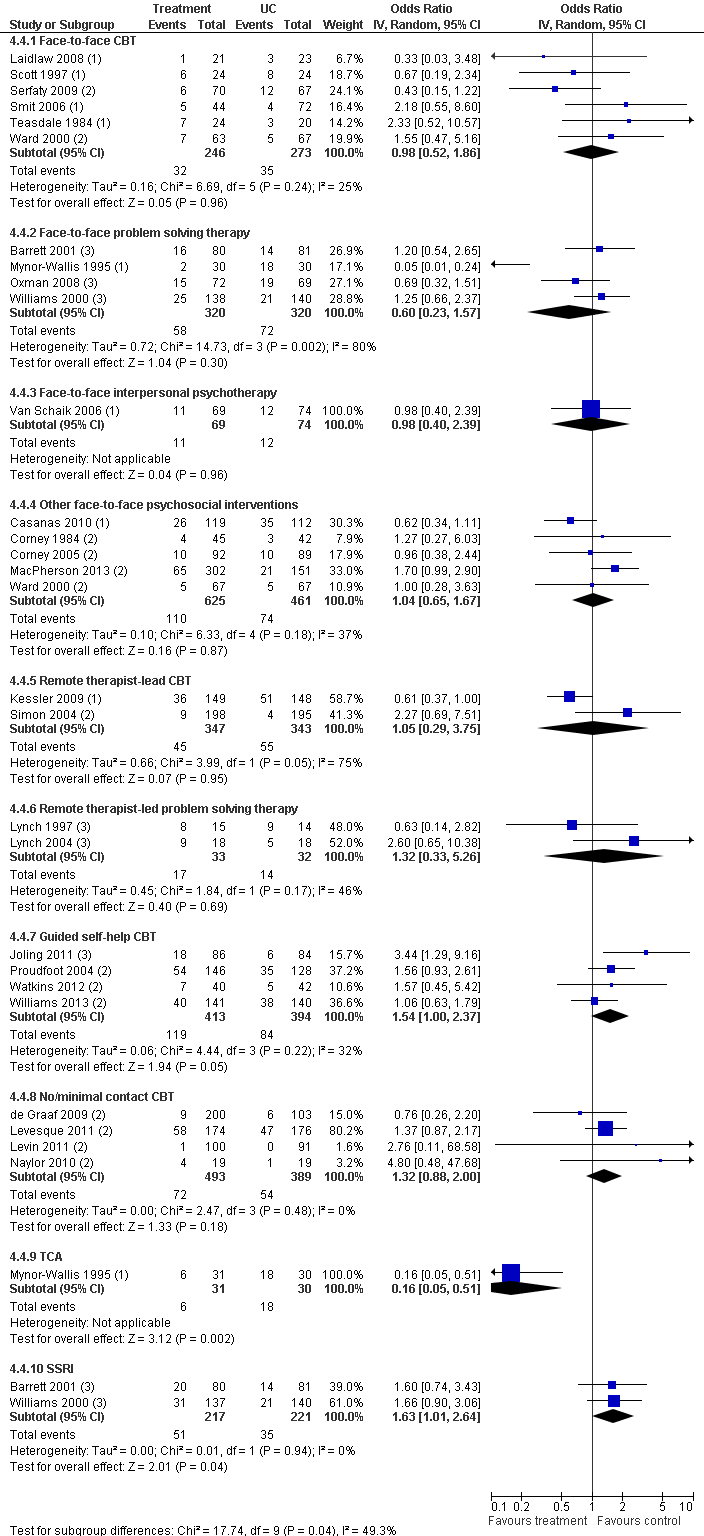


4.5. Outcome: response – Comparison: psychosocial therapies (PST1) vs. psychosocial therapies (PST2)


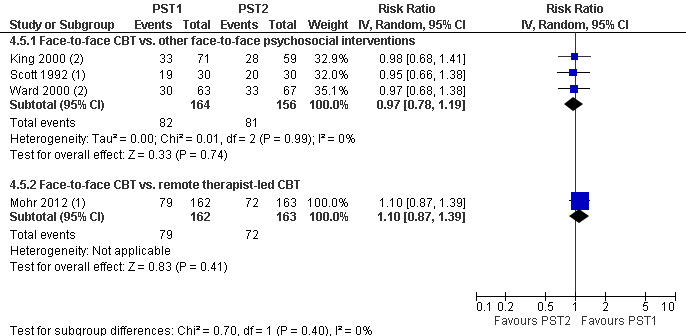


4.6. Outcome: remission – Comparison: psychosocial therapies (PST1) vs. psychosocial therapies (PST2)


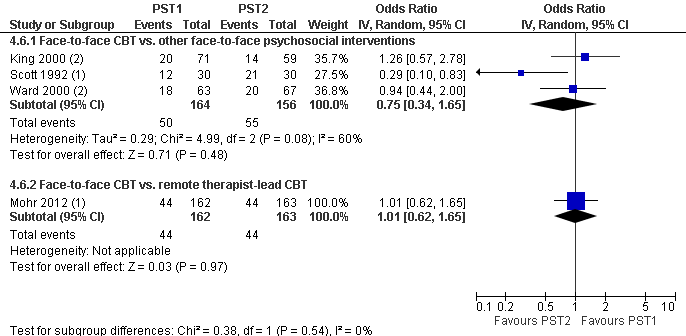


4.7. Outcome: post-treatment depression scores – Comparison: psychosocial therapies (PST1) vs. psychosocial therapies (PST2)


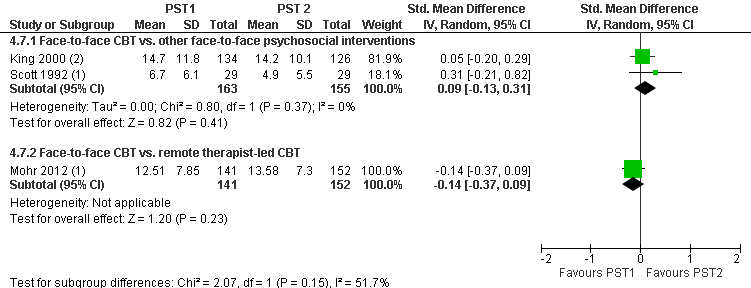


4.8. Outcome: drop-out – Comparison: psychosocial therapies (PST1) vs. psychosocial therapies (PST2)


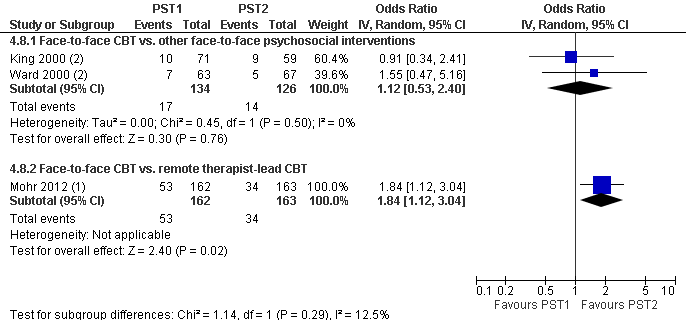


4.9. Outcome: response – Comparison: psychosocial therapies (PST) vs. pharmacotherapy


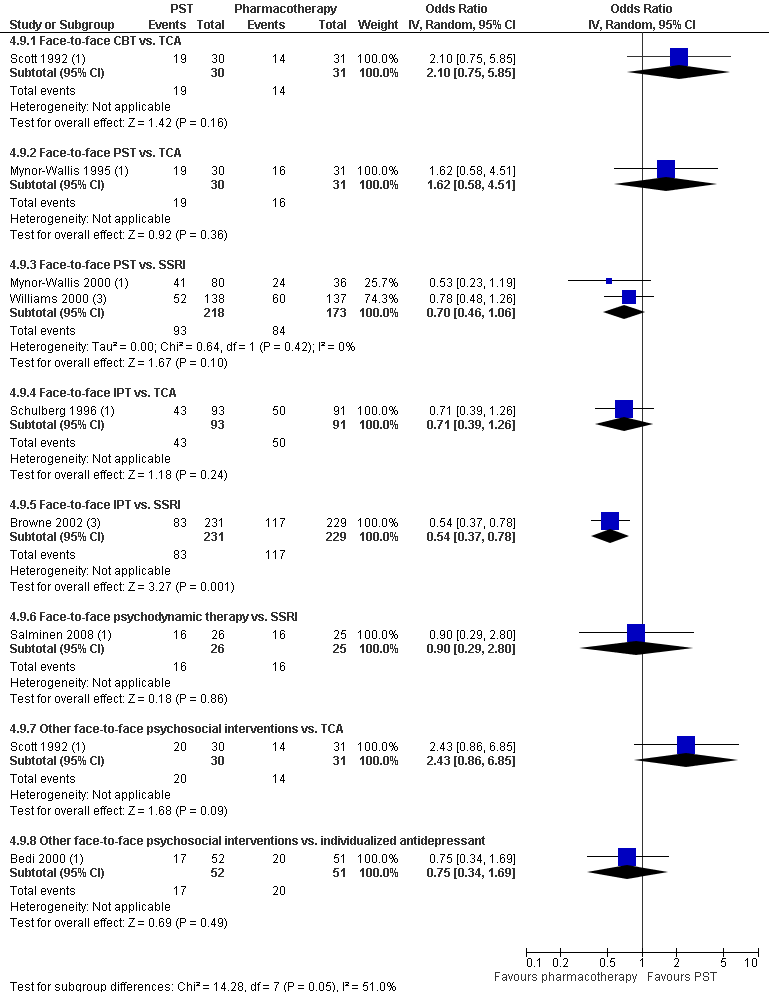


4.10. Outcome: remission – Comparison: psychosocial therapies (PST) vs. pharmacotherapy


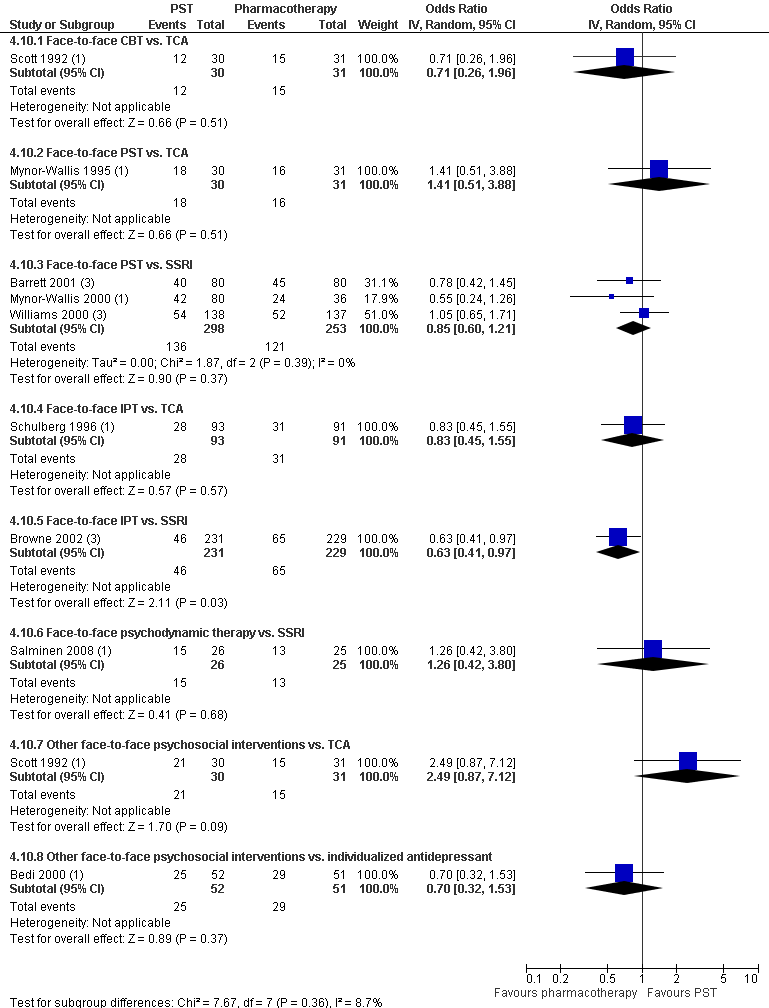


4.11. Outcome: post-treatment depression scores – Comparison: psychosocial therapies (PST) vs. pharmacotherapy


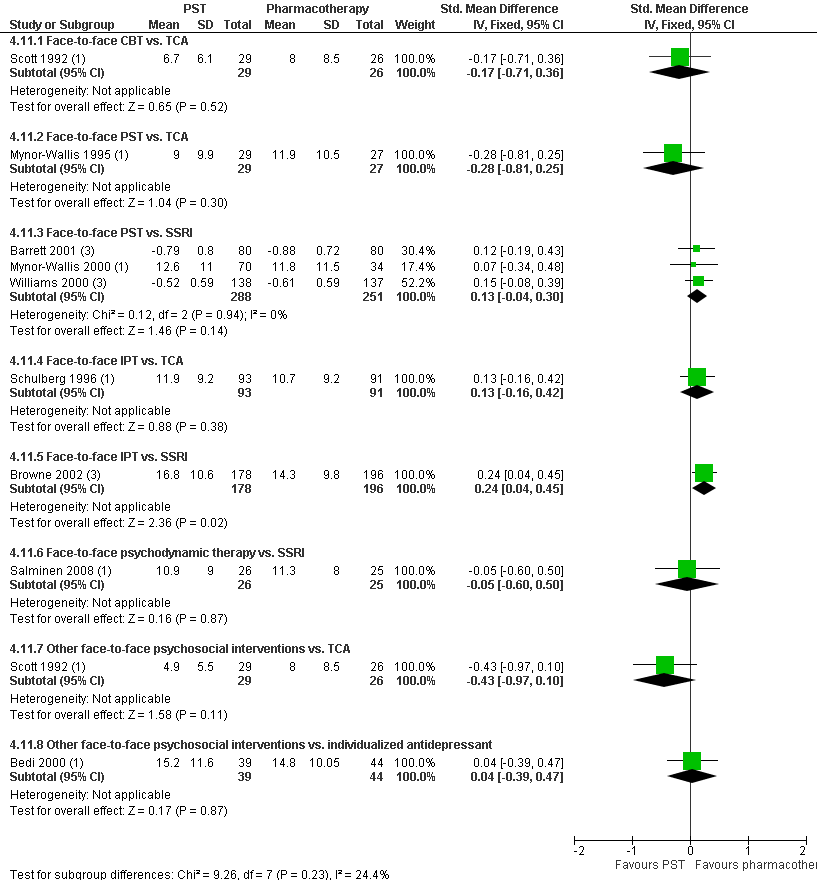


4.12. Outcome: drop-out – Comparison: psychosocial therapies (PST) vs. pharmacotherapy


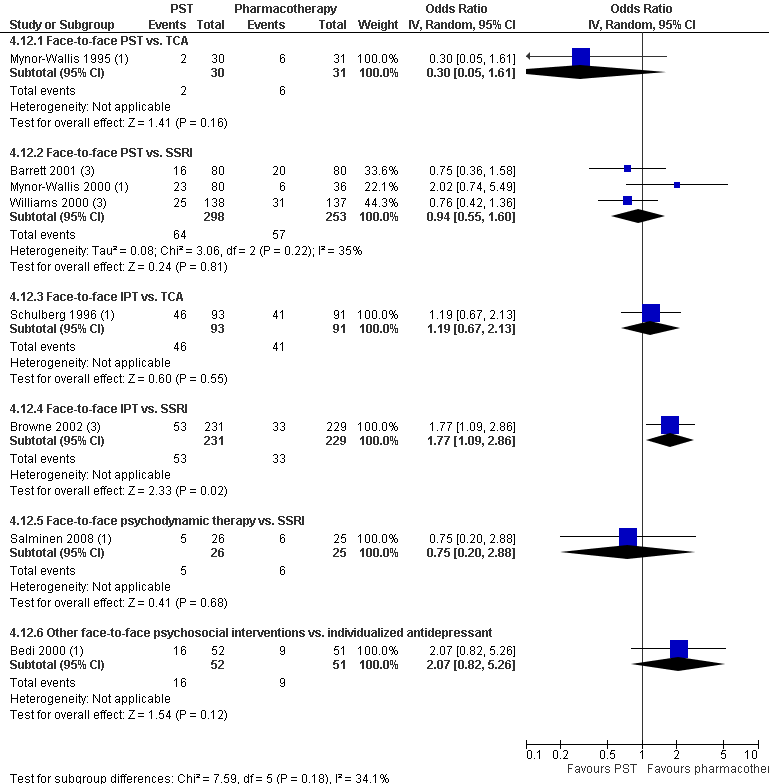


4.13. Outcome: response – Comparison: psychosocial therapies + pharmacotherapy (PST + pharmacotherapy) vs. pharmacotherapy


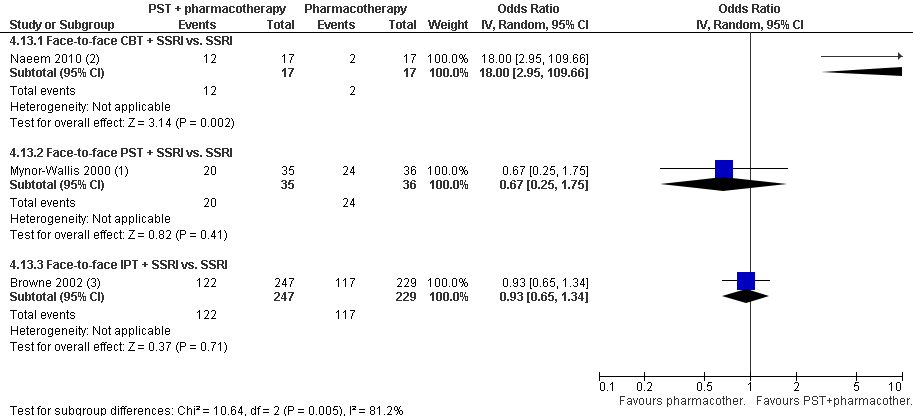


Comment: Response data for Naem 2010 imputed from post treatment depression score means and standard deviations. The publication reports unusually small standard deviations; however, these standard deviations are consistent with very small 95% confidence intervals and p-values reported. Authors did not answer to email inquiry

4.14. Outcome: remission – Comparison: psychosocial therapies plus pharmacotherapy vs. pharmacotherapy


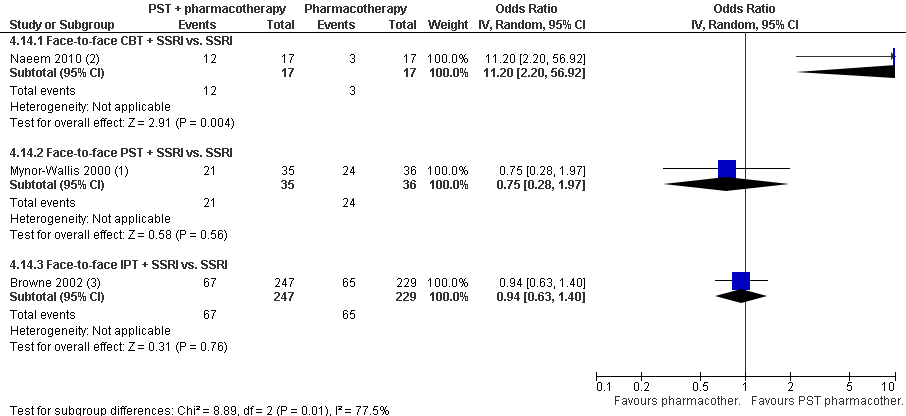


Comment: Remission data for Naem 2010 imputed from post treatment depression score means and standard deviations. The publication reports unusually small standard deviations; however, these standard deviations are consistent with very small 95% confidence intervals and p-values reported. Authors did not answer to email inquiry

4.15. Outcome: post-treatment depression scores – Comparison: psychosocial therapies + pharmacotherapy vs. pharmacotherapy


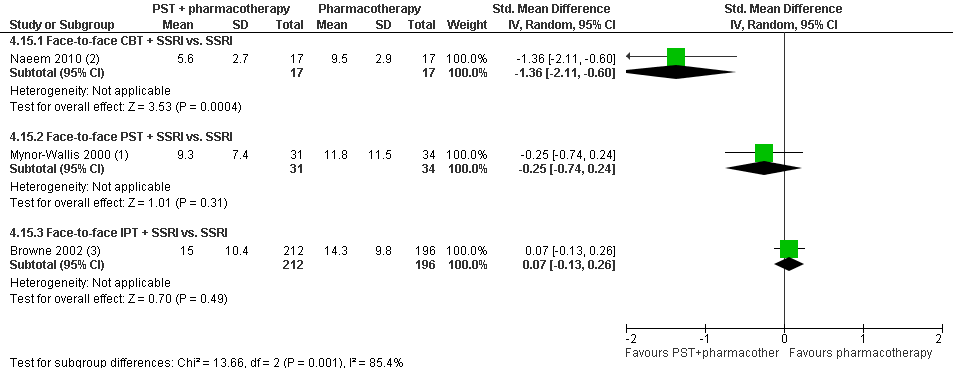


Comment: Naem 2010 reports unusually small standard deviations; however, these standard deviations are consistent with very small 95% confidence intervals and p-values reported. Authors did not answer to email inquiry

4.16. Outcome: drop-out – Comparison: psychosocial therapies plus pharmacotherapy vs. pharmacotherapy


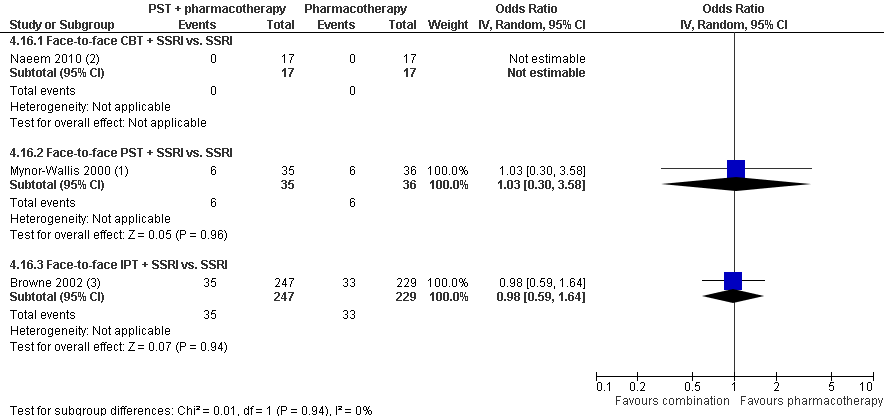


4.17. Outcome: response – Comparison: psychosocial therapies + pharmacotherapy (PST + pharmacotherapy) vs. psychosocial therapies


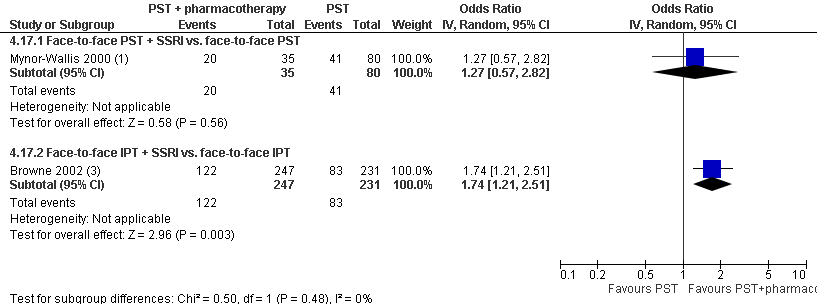


4.18. Outcome: remission – Comparison: psychosocial therapies plus pharmacotherapy vs. psychosocial therapies


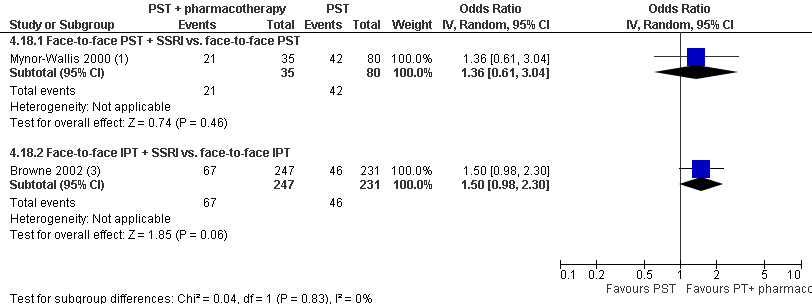


4.19. Outcome: post-treatment depression scores – Comparison: psychosocial therapies + pharmacotherapy vs. pharmacotherapy


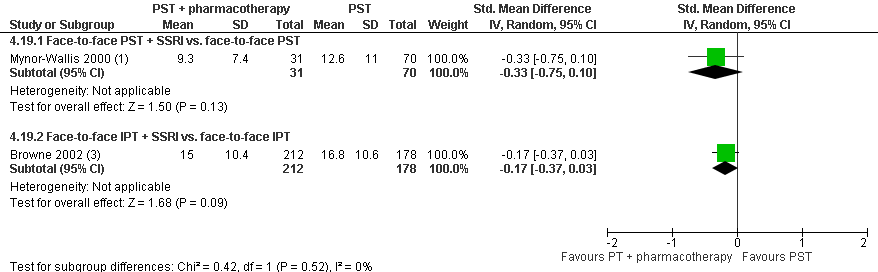


4.20. Outcome: drop-out – Comparison: psychosocial therapies plus pharmacotherapy vs. psychosocial therapies


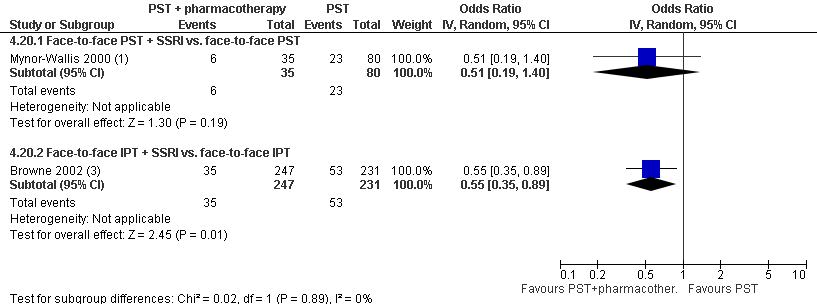


**5. Funnel plots**

(only for comparisons with at least 5 trials)

5.1. Outcome: Response – Comparison: psychosocial therapies vs. usual care


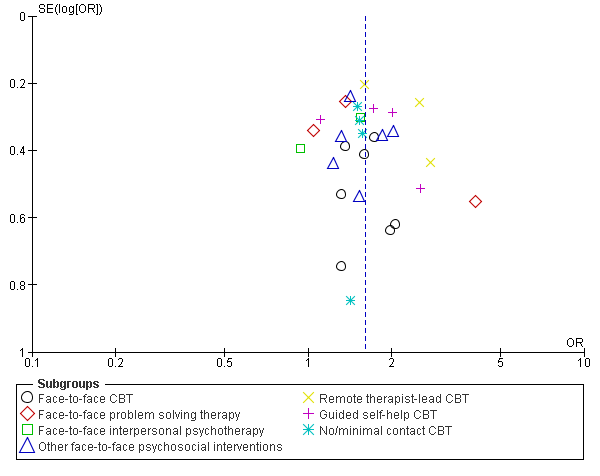


5.2. Outcome: Remission – Comparison: psychosocial therapies vs. usual care


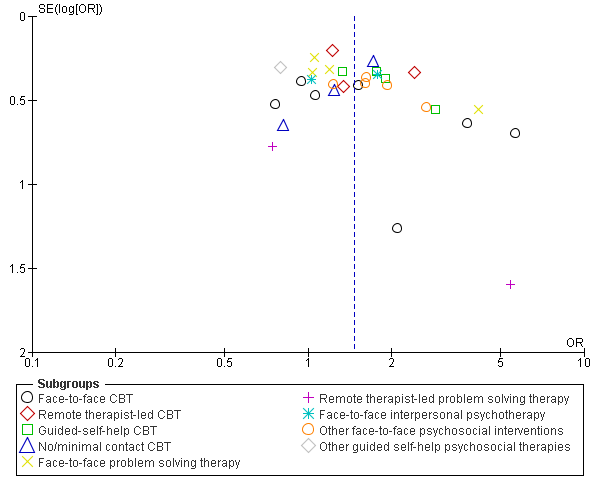


5.3. Outcome: post-treatment depression scores – Comparison: psychosocial therapies vs. usual care


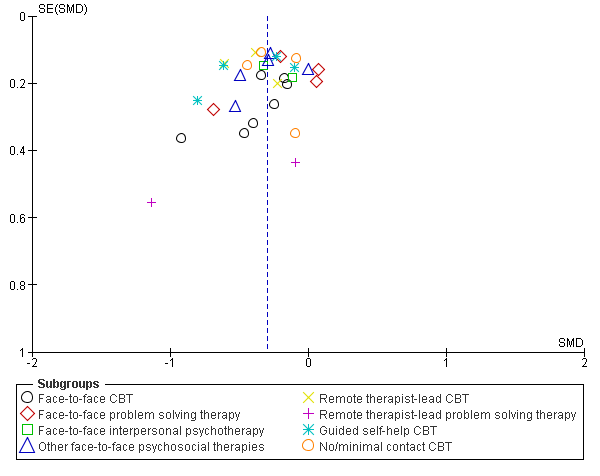


5.4. Outcome: drop-out – Comparison: psychosocial therapies vs. usual care


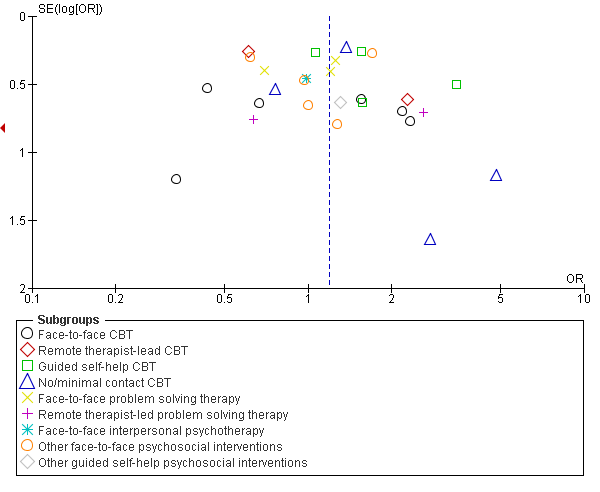


5.5. Outcome: Response – Comparison: psychosocial therapies vs. pharmacotherapy


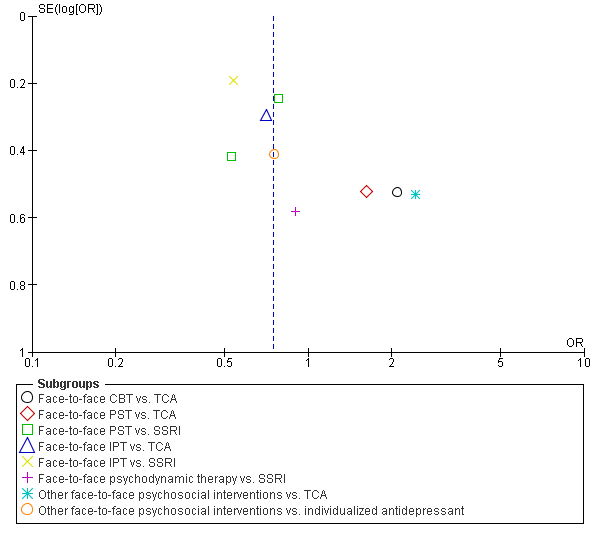


5.6. Outcome: Remission – Comparison: psychosocial therapies vs. pharmacotherapy


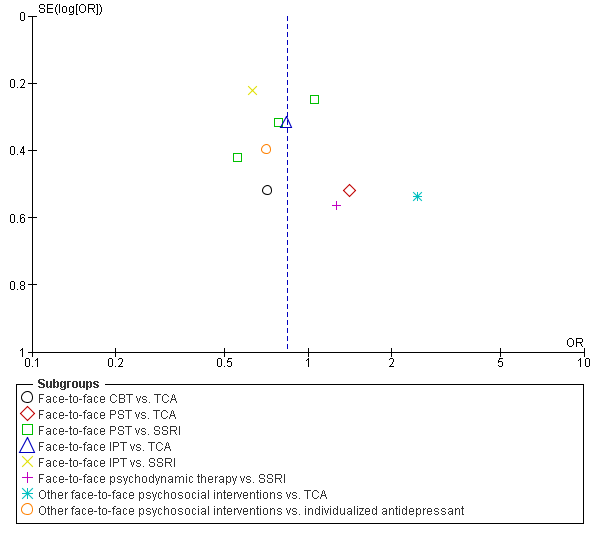


5.7. Outcome: post-treatment depression scores – Comparison: psychosocial therapies vs. pharmacotherapy


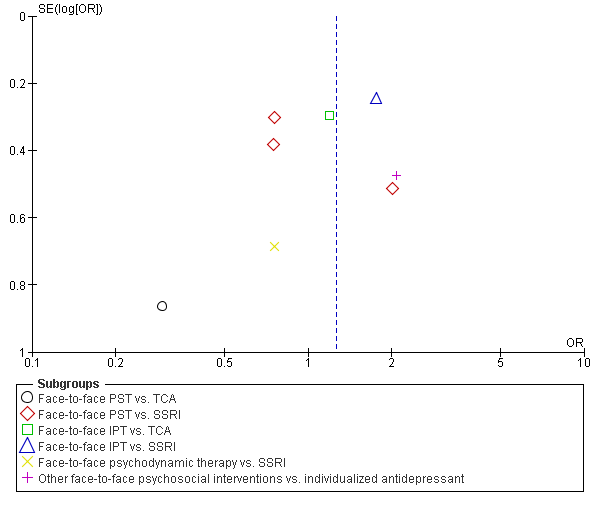


5.8. Outcome: drop-out – Comparison: psychosocial therapies vs. pharmacotherapy


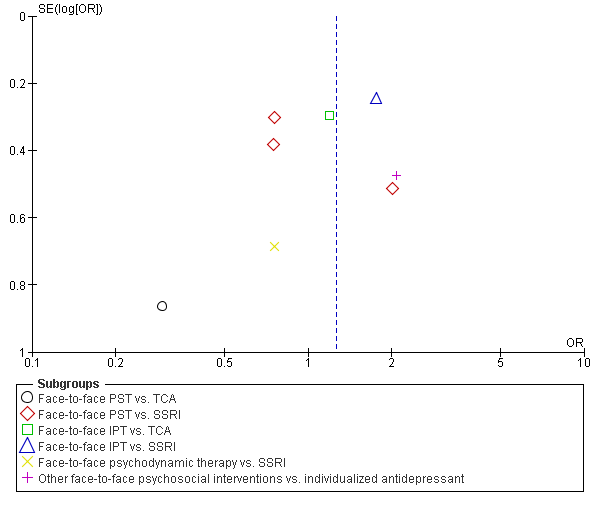


**6. Findings from meta-regression analyses**

Table 6.1.

Outcome response – meta-regression analyses

|  | Regression coefficient | 95% credible intervals |
| --- | --- | --- |
| Duration of treatment | 0.0088 | -0.0187 to 0.0367 |
| Number of sessions | 0.0328 | -0.0313 to 0.0958 |
| Sample size | -0.0005 | -0.0032 to 0.0019 |
| Major depression no/yes | 0.3624 | 0.0072 to 0.7196 |
| Minor depression/dysthymia no/yes | -0.5049 | -1.026 to 0.0208 |
| Age > 55 years no/yes | -0.3097 | -0.7491 to 0.0459 |
| Risk of bias high/unclear/low | -0.0918 | -0.3022 to 0.1044 |
| Recruitment clinical/screening | -0.022 | -0.3166 to 0.2736 |

**7. Net heat plot for investigating inconsistency**

Explanation for the net heat plot, following Krahn et al. (2013):

- The colors on the diagonal represent the inconsistency contribution of the corresponding design.

- The colors on the off-diagonal are associated with the change in inconsistency between direct and indirect evidence in a network estimate in the row after relaxing the consistency assumption for the effect of one design in the column.

- Cool colors indicate an increase and warm colors a decrease: A blue colored element indicates that the evidence of the design in the column supports the evidence in the row. A red colored element indicates that the evidence of the design in the column contrasts to the evidence in the row.

Only two warm spots were detected: Comparison PST:UC within design PST:TCA:UCC contributes some minor inconsistency (Study Mynor-Wallis 1995)
